# Modulation of immune response by nanoparticle-based immunotherapy against food allergens

**DOI:** 10.3389/fimmu.2023.1229667

**Published:** 2023-09-08

**Authors:** Sivadas Swathi Krishna, Syeda Ayesha Farhana, Ardra T.P., Shalam M. Hussain, Vidya Viswanad, Muhammed Hassan Nasr, Ram Kumar Sahu, Jiyauddin Khan

**Affiliations:** ^1^ Department of Pharmaceutics, Amrita School of Pharmacy, Amrita Institute of Medical Science (AIMS) Health Science Campus, Amrita Vishwa Vidyapeetham, Kochi, India; ^2^ Department of Pharmaceutics, Unaizah College of Pharmacy, Qassim University, Unaizah, Saudi Arabia; ^3^ Department of Clinical Pharmacy, College of Nursing and Health Sciences, Al-Rayyan Medical College, Madinah, Saudi Arabia; ^4^ Department of Clinical Pharmacy, Faculty of Health Sciences and Nursing, Al-Rayan Colleges, Al-Madinah Al-Munawarah, Saudi Arabia; ^5^ Department of Pharmaceutical Sciences, Hemvati Nandan Bahuguna Garhwal University (A Central University), Chauras, Tehri, Uttarakhand, India; ^6^ School of Pharmacy, Management and Science University, Shah Alam, Selangor, Malaysia

**Keywords:** allergen immunotherapy, food-induced anaphylaxis, oral immunotherapy, sublingual immunotherapy, nanoparticles

## Abstract

The increasing prevalence of food allergies worldwide and the subsequent life-threatening anaphylactic reactions often have sparse treatment options, providing only symptomatic relief. Great strides have been made in research and in clinics in recent years to offer novel therapies for the treatment of allergic disorders. However, current allergen immunotherapy has its own shortcomings in terms of long-term efficacy and safety, due to the local side effects and the possibility of anaphylaxis. Allergen-specific immunotherapy is an established therapy in treating allergic asthma, allergic rhinitis, and allergic conjunctivitis. It acts through the downregulation of T cell, and IgE-mediated reactions, as well as desensitization, a process of food tolerance without any allergic events. This would result in a protective reaction that lasts for approximately 3 years, even after the withdrawal of therapy. Furthermore, allergen-specific immunotherapy also exploits several routes such as oral, sublingual, and epicutaneous immunotherapy. As the safety and efficacy of allergen immunotherapy are still under research, the exploration of newer routes such as intra-lymphatic immunotherapy would address unfulfilled needs. In addition, the existence of nanoparticles can be exploited immensely in allergen immunotherapy, which would lead to safer and efficacious therapy. This manuscript highlights a novel drug delivery method for allergen-specific immunotherapy that involves the administration of specific allergens to the patients in gradual increasing doses, to induce desensitization and tolerance, as well as emphasizing different routes of administration, mechanism, and the application of nanoparticles in allergen-specific immunotherapy.

## 1 Introduction

Allergy is an immune system response, an IgE-mediated hypersensitivity reaction that is triggered upon exposure to antigens that are known as allergens. Allergens can be found in the form of faunal products (dander and house dust mites), food, drugs, or flora. Among these, food allergens are perilous, which makes food allergies one of the most prevailing conditions that are life-threatening. Food allergy is a common condition that affects up to 10% of the general population (1). Food allergy, a type 1 hypersensitivity reaction, arises when the immune system attacks the allergens, due to the presence of IL-4 in T cells. Following that, the T cells differentiate into Th2 cells. Then, these Th2 cells produce IL-4, IL-5, and IL-13, which leads to the production of IgE (2) (3). Then, during the elicitation phase, the allergen attaches to IgE and couples with FcRI on the surface of the effector cells (mast cells, eosinophils, and basophils), initiating a rapid release of pro-inflammatory mediators such as histamine and leukotrienes, which induce the allergy symptoms (4). In a recent epidemiological study involving 333,200 children in the US, the occurrence of food allergy was found to be 6.7%. The current scenario shows that there is an increasing prevalence of food allergies, both in children and adults, with life-threatening allergic reactions. However, the refrainment of food allergens and the first-line treatment of anaphylaxis with adrenaline symbolize the current standard of care in food allergy (5). In this scenario, allergen immunotherapy (AIT) plays a pivotal role in the treatment of food allergen-induced anaphylaxis, as food avoidance is ineffective, difficult, and could cause a deterioration to patients’ quality of life, with regard to persistent IgE-mediated food allergies. Even though AIT is a curative, permanent curation, it is difficult to achieve. The main aim of FA-AIT (food allergen-allergen immunotherapy) is sustained unresponsiveness that would help patients to consume normal food without any allergy exposure (6).

The disadvantages of older approaches of treatment are not much effective due to lower bioavailability. The use of nanoparticles has produced sensitive, cost-effective, time-consuming, and selective methods that can replace conventional methods used in recent years. However, each nanomaterial has demonstrated a distinct potential for certain allergens or classes (7). It helps to design novel allergen immune therapy strategies. Thereby, it has an significant impact on the safety, efficacy on the food allergen induced allergic reaction (8). In addition, allergen-delivery systems based on nanoparticles are now possible because to advancements in nanotechnology, which can be used as potential adjuvants in allergen-specific immunotherapy. Since of their improved bioavailability and focused distribution of therapy molecules, the use of nanoparticles offers the possibility of a remedy for allergic reactions that is more effective than other methods now in practice. Consequently, nanotechnology-based allergen delivery strategies have the goal of developing an innovative and potentially fruitful strategy for allergy immunotherapy (9).

Recent years have seen a sharp increase in the number of individuals who are affected by food allergies, despite the fact that immunotherapy still has numerous significant drawbacks that require to be corrected. These negatives include longer intervention periods (months or years), prevalent hospital, significant expenditures, a greater likelihood of adverse events throughout therapy, and a reduction of sustainability in desensitization. Additionally, there is a higher chance of adverse events during therapy. This paper discusses the recent discoveries in innovative allergen-specific treatments for food allergy, as well as a synopsis of food allergy grouping, the mechanism involved in immune response, as well as the advancements in research and unique delivery breakthroughs.

## 2 Food allergy

Food allergies are generally categorized as IgE-mediated food allergies, mixed food allergies (mediated by IgE-dependent and IgE-independent mechanisms) and non-IgE-mediated food allergies, based on the type of mediators involved (2). Some of the major food allergens with their allergenic constituents are listed in the given table (**Table 1**).

**Table 1:** List of a few major food allergens with their allergenic constituents.

Food	Threshold level (mg)
Egg white Egg yolk	2.9 (Pasteurized whole egg) 0.13 (Whole raw), 0.2 (raw white), 10 (cooked white)
Cow’s milk	15 mg
Wheat	15 mg
Peanut	0.25 (Ground), 1.25 (crushed)
Crustacean shellfish	Not reported

### 2.1 IgE mediated food allergy

According to an increasing body of research, IgE antibodies and mast cells may serve not just as effectors of acute hypersensitivity, but also as amplifiers during first antigen exposure (10). When an individual gets exposed to a food allergen, this exposure induces an initial immune response. On subsequent exposure to the same allergen, it triggers IgE mediated response with the involvement of mast cells and basophils that results in an immediate expression of symptoms. Crosslinking takes place as a result of the attachment of allergen-derived epitopes to IgE molecules. This crosslinking is what causes the release of prepared inflammatory mediators like histamine. This results in the appearance of allergic responses, which is then followed by the creation and release of leukotrienes, platelet-activating factors, and other cytokines that continue to perpetuate the allergic inflammation. The gastrointestinal tract (GIT), the skin, and the respiratory system are the primary organs that are impacted by this kind of allergy, and it frequently causes systemic symptoms. This is the most frequent kind of condition that can be fatally allergic to food (11). IgE-mediated food allergies develop when the essential immunological components that support tolerance and avoid innocuous food antigens from being mistakenly identified as pathogens lose their integrity. Clinical symptoms typically appear minutes to hours after consumption, which typically have a quick onset. Additionally, it raises the possibility of adverse or deadly reactions. While atopic reactions to peanuts, tree nuts, and shellfish typically last into adulthood, IgE-mediated allergies to cow’s milk, egg, wheat, and soy are more likely to be outgrown.

The food antigen crosses the mucosal barrier and get processed by the dentritic cells. This process results in the activation of dentritic cells, which in turn stimulates naive T cells to develop a T helper cell 2 (Th2) phenotype. This stimulates inflammatory signals that lead to the production of food antigen-specific IgE by food Ag (antigen)-specific B cells, hence fostering a state of sensitization and allergy. IgE that is specific to a food antigen binds to basophils’ and mast cells’ FceRI (high-affinity IgE receptor) receptors. When exposed to an antigen, mast cells and basophils’ IgE and IgE receptors cross-link, releasing prepared mediators (histamine, tryptase, platelet activating factor, prostaglandins, and leukotrienes) into the bloodstream and hastening the onset of symptoms.

Gastrointestinal manifestations can include oral tingling, pruritus, swelling, nausea, abdominal pain and vomiting. Respiratory effects include wheezing and airway inflammation. Skin manifestations include flushing, urticaria, angioedema and pruritus. Systemic responses may also occur, such as hypothermia.

Variants of IgE-mediated food allergy include oral allergy syndrome (OAS), in which individuals with allergic rhinitis produce IgE molecules that are crossreactive with fruit, or vegetable-protein epitopes. Another variant of IgE-mediated food allergy occurs in individuals who produce IgE antibodies that are specific for the red meat carbohydrate galactose-α-1,3-galactose, rather than specific for a protein epitope (5, 12).

### 2.2 Mixed food allergy

Atopic symptoms include things like delayed food allergy-associated atopic dermatitis (6–48 hours after exposure), that is put by the presence of T helper 2 (Th2) cells, and eosinophilic gastrointestinal illnesses like eosinophilic esophagitis (EoE). Milk allergies and the eosinophilic infiltration of tissues brought on by IgE-independent mechanisms are common triggers for these reactions (13). Currently, research is being conducted to identify how the IgE-mediated and non-IgE-mediated pathways augment food-induced anaphylaxis (14). Some of the examples of mixed food allergies include atopic dermatitis, asthma, eosinophilic gastroenteritis, eosinophilic gastritis, and eosinophilic esophagitis. Mixed food allergies are frequently implicated in causing gastrointestinal symptoms in children and adults, but they most likely will remain underdiagnosed, as the evidence-based protocols needed to diagnose and treat these diseases are still lacking (15).

### 2.3 Non-IgE-mediated food allergy

Non-IgE-mediated food allergy is defined as an immune reaction that occurs against the food, but not via the synthesis of IgE, and it does not activate the mast cells or basophils (16). This allergy is mediated by allergen-specific T cells that primarily affect the GIT and cause food protein-induced enterocolitis syndrome (FPIES), food protein-induced proctocolitis (FPIP), and food protein enteropathy (FPE). This type of allergy is also known as a sub-acute or chronic inflammatory process (17).

The food protein-induced allergy proctocolitis of infancy, food protein-induced enterocolitis syndrome (FPIES), pulmonary hemosiderosis (Heiner syndrome), and celiac disease are all examples of non-IgE-mediated reactions. Non-IgE-mediated food allergies are immunologic reactions to food that take place in the absence of conspicuous IgE antibodies that are specific to the food, in the skin or serum, and as a result, they possess a variety of distinct pathogenetic pathways. Non-IgE-mediated reactions begin slowly, and they are mostly T cell-driven, with the involvement of other cells, as well as macrophages, eosinophils, or neutrophils.

Lethargy, pallor, and frequent excessive vomiting are typical acute symptoms of FPIES, which often appear 1 to 4 hours (mostly 2 hours) after consumption. Rice and milk are frequently mentioned as FPIE triggers, followed by soy, oats, eggs, and poultry. Presently, a biomarker or *in vitro* test are unavailable for the diagnosis of FPIES or the food allergy that it causes, as there is no concrete evidence that suggests which cell is ultimately responsible for antigen identification and reaction initiation (17).

## 3 Mechanism of the immune response

The immune system is categorized into two parts: innate and adaptive immunity. Innate immunity is a nonspecific immune mechanism that occurs rapidly upon exposure to antigens in the body. The innate immune response is mediated by the skin as a barrier, chemicals in the body, and the immune cells. Whereas, adaptive immunity is an antigen-specific immune mechanism that is more complex than an innate response. The immunological response is chiefly dependent upon the T lymphocytes, also known as T helper cells—Th1 and Th2. The TH1 cells trigger the release of variant chemical mediators as a first-line defense mechanism against invasion from harmful pathogens in normal individuals. Whereas, in an atopic individual, Th2 cells with their mediators uplift the immunological system to recognize the food allergens as an invader and produce a response against these allergens (18). ILC2 is type 2 innate lymphoid cells. It is a type of innate lymphoid cell, which is derived from lymphoid progenitor and belongs to the lymphoid groups. They are subsets of lymphoid cells. These cells lack recombinant activating gene and lacks B and T cell receptor. ILC2 secretes large amounts of IL-5, IL-9 and IL-13. ILC2 plays an important role in allergic reactions. These cytokines can activate the eosinophils, mast cells and plays a role in food allergen immune response. ILC2 resides in the mucosal tissues. They mainly lack lineage markers. They secrete large number of cytokines, activates immune cells and cause pathologic as well as physiologic changes.

In case of food allergy IgE mediated is the most common. In this scenario ILC2 plays an important role. After allergic sensitization, IL-25 and Il-2 obtained from CD4+ cells Th2 cells stimulates ILC2 and produce Il-13 which increases IgE mediated food allergy (19, 20). It mediates the immunosuppressive effect. It acts by activation of eosinophils and IL-5 through an independent pathway. ILC2 plays a role in homeostasis and helps in transition from innate to adaptive immunity (21, 22)

An individual undergoes allergen sensitization, resulting in an allergic response. The allergens are detected by antigen-presenting cells (APCs) such as macrophages and dendritic cells, which are abundantly present on the mucosal surface of the body. These allergens that interact with the APCs are subsequently absorbed and presented on the major histocompatibility complex class II (MHCII) to T cells. This helper T cell differentiates into Th2 in the presence of IL-4, which releases inflammatory markers such as IL-4, IL-5, IL-10, and IL-13. This leads to a massive production of IgE by B cell-derived plasma cells. Then, these allergens bind to the high-affinity IgE receptor (FcϵRI) that is present on mast cells and basophils. This is followed by the production of IL-25, IL-33, and TSLP by the epithelial cells, which further affect the Th2 response (23). Consequently, they would release IgE with the expansion and differentiation of Th2 cells (24).

**Figure 1:**
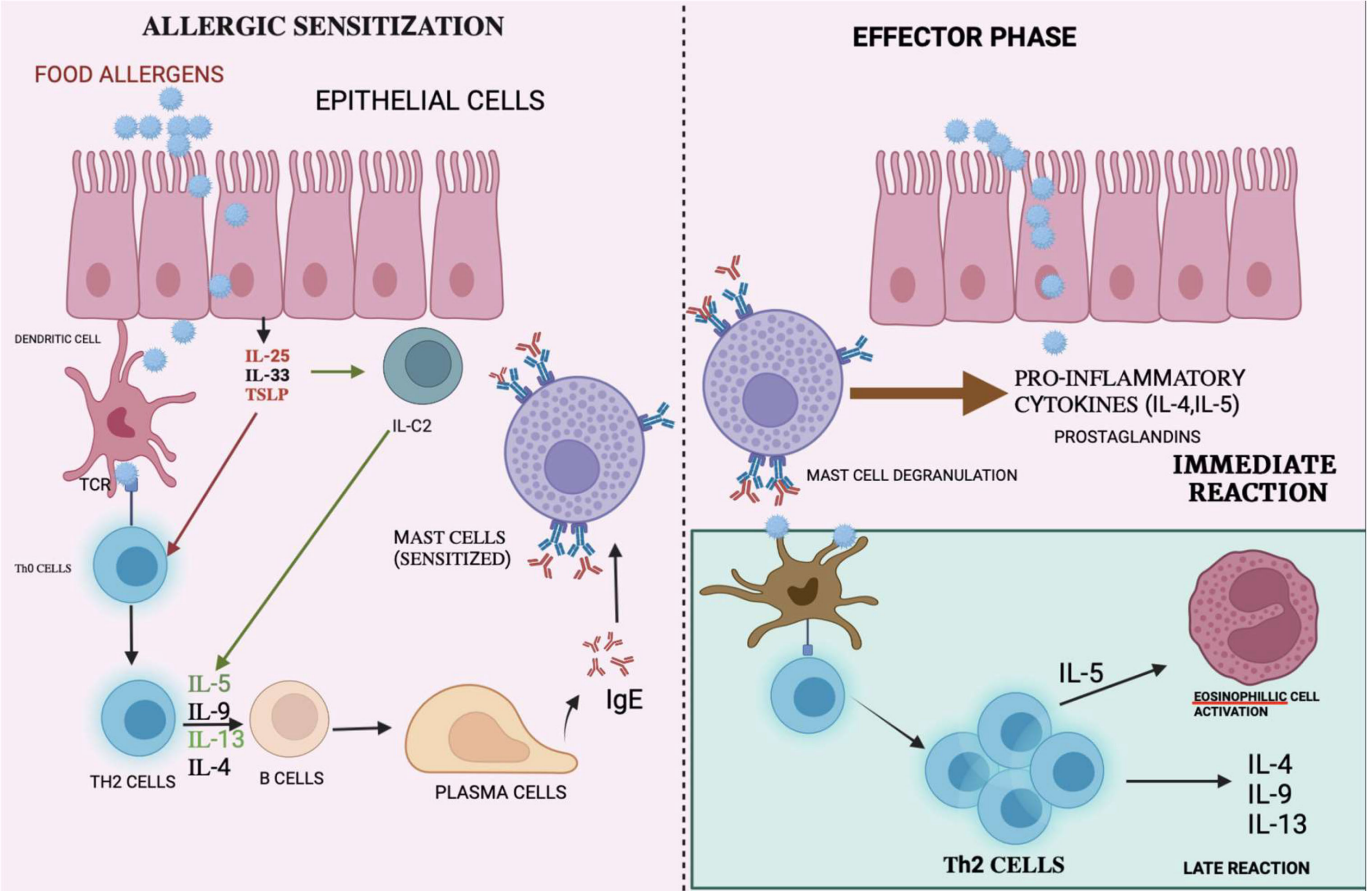
Mechanism of immune reactions: Allergen are presented to the T-cells by APC causing differentiation of T-cells to T-helper cells. The interaction with interleukins causes the plasma cells to release IgE which binds on mast cells causing mast cell degranulation thereby releasing pro-inflammatory cytokine, which causes immune reactions.

Once released, IgE binds to the high-affinity receptor on the surfaces of mast cells and basophils. Upon subsequent exposure to the same allergen, the allergic reaction is stimulated as described above, which leads to degranulation, followed by the release of inflammatory mediators such as histamine, prostaglandins, leukotrienes, and other cytokines. Therefore, this would cause hypersensitivity reactions that affect different organs including the GIT, skin, and the respiratory system (25). The mechanism of immune response is shown in **Figure 1**.

The immune mechanism of food allergy involves the tolerance, sensitization and desensitization.

### 3.1 Tolerance

The healthy immune system becomes tolerant to food, which can be characterized as unresponsiveness towards potential food allergens. Immune tolerance is an active condition that begins with the uptake of food antigens in the small intestine, which is filled with gut-associated lymphoid tissue (GALT) (26). In a set of actions that would sustain immune tolerance, various cell types function together to transfer antigens from the gut lumen to the lamina propria. The lymphoid tissue present antigens, which trigger a T-cell response in the lymphoid tissue, and revert the immune effector cells to the gut. Paracytosis and transcytosis are the two mechanisms by which food antigens can be captured from the gut lumen. Dendritic cells (DCs) and/or macrophages, which are myeloid cells, are able to capture potential dietary antigens from the gut lumen. Specialized cells such as the M (microfold) cells, situated near the GALT, would be able to endocytose the antigen (27). CD103+ DCs, for example, is an antigen that can pass the epithelial barrier through transcytosis or translocation with the help of mucin-secreting goblet cells. It captures the antigen from the lumen, either through a tight junction, or by creating transcellular pores in M cells. A chemokine receptor CX3C extends dendrites between the epithelial cells through the secretion of IL-10. The CD103+ DCs would also produce transforming growth factor-β (TGFβ) and retinoic acid during migration from the lamina propia to the lymph nodes. CD103+ DCsmay also induce the differentiation of naive CD4+ T cells into FOXP3−, IL-10-secreting T cells, and possibly type 1 regulatory T cells (Tr1 cells). The movement of these cells to the lymph node can improve tolerance towards food allergens. Furthermore, the retinoic acid produced during the migration can also induce Treg cell expression of integrin α4β7. Human clinical trials demonstrating hypomethylation of the FOXP3 locus of T reg cells in those individuals who establish and maintain functional tolerance in response to oral immunotherapy, have provided the necessary information regarding the role of Treg cells in oral tolerance (OIT) (28).

### 3.2 Sensitization

When APC in the gut mucosal epithelial cells come in contact with the allergen, they recruit dendritic cells and convert them into pro allergic phenotype (DC2). DC2 takes up the allergen molecules and move into the lymph nodes, present it to the naive T cells and develops Th2 and TFH subsets. These subsets promote maturation of B cell which leads to allergen-specific IgE production through class-switch recombination. The produced IgE molecules binds to the surface of basophils and mast cells through high affinity receptors thereby sensitizing the individual. When the sensitized individual is re exposed to the allergen, it causes the crosslinking of bound IgE molecules, degranulation of mast cells and release of vasodilatory and chemoattractive mediators (29).

### 3.3 Desensitization

The exact mechanism of desensitization in food allergy is still unclear. Desensitization of mast cells and basophils mainly occur due to anaphylaxis during immunotherapy. It mainly occur after first administration of immunotherapy agents. It has been hypothesised that in food allergy the desensitization occurs through same mechanism as the exact mechanism is lacking.

There is evidence that desensitization to MC is both allergen-specific and reversible. Increasing dosages of allergen can cause IgE-FcRI complex internalization, which makes mast cells insensitive to allergen stimulation. This is one of the molecular mechanisms. Internalization of the TgE-FcRI complex, reduced calcium-paired calcium flow in mast cells, and dysregulation of the STAT6 pathway are some of the proposed mechanisms of desensitization. There is evidence from a variety of *in vitro* experiments to support the hypothesis that increasing dosages of allergen cause IgE-FcRI complex internalization in mast cells, which in turn renders MCs resistant to allergen challenge. Desensitization is successful whether there is a complete or partial reduction of IgE in the patient (30).

## 4 Management of food allergy: Current insights

Food allergy is a global health concern that has caused a tremendous reduction in the quality of life of those affected. The current paradigm is a stringent avoidance of food allergens and self-management through the usage of epinephrine auto injectors during an emergency. However, it is crucial to acknowledge that accidental exposure to food allergens can result in life-threatening anaphylaxis (25). Additionally, antihistamines, corticosteroids, and oxygen treatment have been undertaken for the mitigation of allergic symptoms (31). Presently, several treatment approaches are under investigation, which comprise allergen-specific immunotherapy and allergen-nonspecific approaches.

Allergen-specific approaches include the desensitization of atopic individuals with food allergens via oral, sublingual, and epicutaneous immunotherapy; the adoption of recombinant proteins, and a paradigm of an extensively heated diet containing milk and egg (5). Allergen’s nonspecific approach is mediated by monoclonal IgE antibodies and the use of Chinese herbal formulations, which are still under clinical trials (32).

### 4.1 Allergen-specific immunotherapy

Allergen immunotherapy, which is also known as allergen shots, focuses on the administration of gradually incremented doses of allergen that render two possible payoffs: desensitization and tolerance. Desensitization is defined as transitory hypo-responsiveness, owing to an unceasing allergen exposure that would ultimately end up with an increased threshold of reactivity to the food allergen. Tolerance, on the other hand, is the ability to consume allergic food even after the withdrawal of therapy (33).

#### 4.1.1 Mechanism of AIT

**Figure 2:**
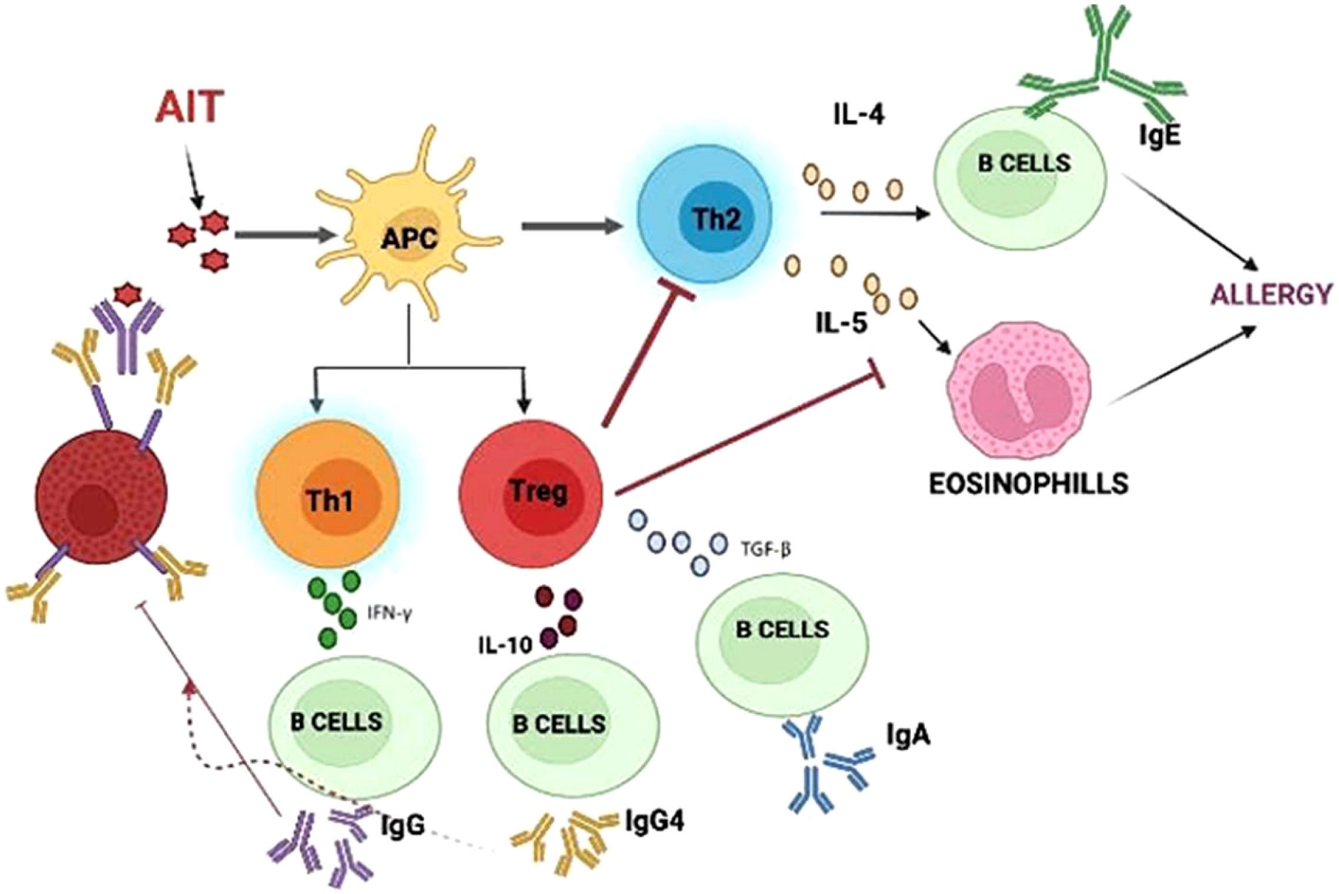
Mechanism of Allergen immunotherapy: AIT molecules interact with APC causing regulatory cells to inhibit further steps of immune response.

AIT is mediated through immunomodulation, which affects the T lymphocytes. The findings have suggested that there is an increase in CD8+ cells, along with an increased production of IL-4 and IFN-γ, which may be a result of an increased TH1 response or the downregulation of Th2 cells. Moreover, the generated Treg cells would inhibit the cytokine response (34). There is also a marked reduction in basophils and eosinophils that ultimately lead to a declined IgE-mediated release rate of histamine. During the initial course of AIT, an elevated serum concentration of IgE is observed, which gradually declines during therapy. An immense increase in the level of IgG-blocking antibodies can be interpreted as successful AIT. The production of IgG2a, IgG2b, and IgG3, and the synthesis of IgG1 are associated with the Th1 and Th2 responses, respectively (35). The activation of IgE is suppressed by the action of IgG through interaction with IgE before it can crosslink with the IgE receptors on mast cells and basophils (36), as shown in **Figure 2** below.

#### 4.1.2 Routes of administration

Allergen immunotherapy is an effective strategy to treat food allergies, despite questions regarding the clinical safety and the unorthodox method of delivery in AIT. Current research, however, is focusing on alternative routes and delivery methods that are safer and more convenient for supplying the allergenic extracts. AIT comprises three major forms of treatment, upon which research is focused:

1. Oral immunotherapy (OIT);
2. Sublingual immunotherapy (SLIT);
3. Epicutaneous immunotherapy (EPIT).

The alternative routes include subcutaneous, transcutaneous, intranasal, and intra-lymphatic immunotherapy.

##### 4.1.2.1 Oral immunotherapy

Oral immunotherapy (OIT) has been subjected to clinical trials over the last few years, and it is believed to be a potent therapy for food allergies. OIT is associated with the consumption of steadily incrementing doses of allergen extract that are usually combined with a vehicle. Several OIT protocols exist, which primarily depend upon the dose and the type of food (milk, peanut, egg, etc.). These food allergens can be obtained from any ordinary grocery stores. This, however, would raise a significant query regarding the safety and quality of the therapy. Therefore, regulated standards and guidelines from the Food and Drug Administration have been set up to ensure the safety and quality of the therapy (37).

The maximum OIT protocol is a three-phase program involving:

i. *Initial escalation phase*—This phase is characterized by a dose escalation on the first day of therapy, with 6 to 8 doses of allergenic extract, starting from the very smallest dose, typically 0.1 mg, which is gradually incremented to a maximum of 25 mg. This phase is designed to establish a sub-threshold level and maintenance.
ii. *Build-up phase*—The dose would vary significantly in escalation, which is typically monitored by a physician over weeks to months.
iii. *Dose maintenance—*The dose and the period of treatment would differ between different studies, with a huge increment in the dose, up to 4000 mg, and a range from months to years (38).

###### 4.1.2.1.1 Immunological changes

The principal mechanism is not completely understood, and it is still under investigation. Based on several studies conducted, a significant increase in the food-specific IgG4 antibody was observed, with a decline in basophil and mast cell activation and response. After a few months, there was a deviation observed from the Th2 to the Th1 profile. A heightened action of CD4+ and CD25+ cells were observed, together with the suppression of the immune system by regulatory T cells (39, 40).

###### 4.1.2.1.2 Efficacy and safety profile

Shreds of evidence from recent clinical trials have indicated that OIT would lead to desensitization and clinical tolerance among food-allergic patients. However, there is a paucity of sustained unresponsiveness (SU), which is defined as no increment in reactivity during the oral food challenge (OFC). Moreover, the scarcity of long-term follow-up data of the patients discharged is another concern to consider. It is also evident that most of the discharged patients have terminated the intake of allergic food, due to gastrointestinal adverse effects (37).

Adverse events are frequent during OIT, which are similar to all food types. Oral pruritus is the most common local reaction, which is mild and requires either no treatment or antihistamine therapy. Severe gastrointestinal symptoms such as abdominal pain, vomiting, nausea, and occasional reflux would lead to a discontinuation of OIT in most patients. Some cases with severe symptoms, however, may be associated with exercise, infection, hormonal changes during menstruation, or allergen co-exposure. Approximately 36% of therapy withdrawal is said to be due to adverse events, in which intolerable GI symptoms have contributed significantly. Moreover, eosinophilic esophagitis has been reported as well, through meta-analysis (41).

##### 4.1.2.2 Sublingual immunotherapy

Sublingual immunotherapy (SLIT) involves the sublingual administration of the allergenic extract in gradually incrementing doses to achieve desensitization and tolerance (42). SLIT exhibits a much safer profile compared to OIT, but it has been found to be less efficacious than OIT. In this strategy, the allergenic extract can be used in the form of drops, tablets, or lyocs (43, 44). The allergenic extract is administered sublingually and must be held beneath the tongue for a few minutes. It can either be spit out or swallowed afterwards. The dose usually falls within a range of 1–10 ug of allergenic extract, which is smaller than in OIT. SLIT exploits the antigen presenting cells (APCs—Langerhans cells), which results in desensitization and tolerance. SLIT, however, is not presently advised in the treatment of food allergies, as it is still under investigation due to the exhibition of desensitization in clinical trials (45). This therapy also follows the dose-escalation and build-up phases.

###### 4.1.2.2.1 Mechanism of SLIT

Oral mucosa is the site application of SLIT. It is rich in APCs and acts as the principal key in therapy, inducing tolerance. Several mechanisms of tolerance have been suggested which include:

i. Lack of mucosal-associated lymphoid tissue (MALT);
ii. Limited number of inflammatory cells (basophils, eosinophils, and mast cells);
iii. Existence of lamina propria-limiting antigen absorption;
iv. IgA secretion restricting antigen penetration;
v. Interferon-Ɣ-producing Th1 lymphocytes and regulatory T cells invoking immunosuppression that is mediated by cytokine release (IL-10) (46).
vi. Presence of Pru p 3 and Ara h 9 proteins (47).

The epithelial tissue, lamina propria, and submucosal layer of the oral mucosa are abundant with APCs, particularly the dendritic cells, which play a pivotal role in the tolerogenic mechanism. Antigens at the site are snatched by the dendritic cells within half an hour, and drift to the regional lymph nodes, with the degradation of the antigen into fragments that are presented to the T cells.

In a retrospective comparative study between OIT and SLIT among food-allergic patients, it was evident that patients who received SLIT had rendered a low-threshold dose and faced difficulties in OFC. This indicated that SLIT was less efficacious than OIT, but exhibited a safer profile. SLIT also was found to be more effective among children than adults. The majority of adverse events were confined to local reactions, such as oro-mucosal pruritus being cleared up without any treatment. Furthermore, there were no systemic side effects reported (48).

##### 4.1.2.3 Epicutaneous immunotherapy

Epicutaneous immunotherapy (EPIT) utilizes the novel delivery of antigen that explores the administration of an allergen-containing epicutaneous patch to stimulate the Langerhans cells abundant in the epidermis layer of skin that ultimately would downregulate the effector cell responses. EPIT usually offers a better safety profile with a diminished risk of adverse events associated with the non-vascularized nature of the epidermis. EPIT has been subjected to research, as it presents a promising platform for immunotherapy, with proven efficacy and a self-administrable form (49).

###### 4.1.2.3.1 Immunization, safety and efficacy of EPIT

Epicutaneous therapy primarily aims at the epidermal layer of skin that is described by some salient features: the barrier function by keratinocytes, and the scrutiny of immunity by keratinocytes, Langerhans cells, and non-vascularized nature. Langerhans cells (LCs) are involved in portraying the cellular immune response, whereas the dendritic cells (DCs) in the dermis regulate the B-cell response. The Langerhans cells as APCs will effectively present the antigen and increase the CD8+ cells, while the dendritic cells are involved in the induction of IgA. IL-10 and IL-4 have been found to be secreted with the activation of LCs eliciting Th2-type responses. On the other hand, the DCs(dendritic cells) induce pro-inflammatory cytokines and ultimately, the TH1 response. According to recent studies, it was observed that different types of epithelial cell damage stimulate different molecular pathways that induce the secretion of specific cytokines that portray the immune response, both innate and adaptive. Less intensive epithelial cell damage (abrasion) could also trigger the release of cytokines such as IL-25 and IL-33, which in turn would induce the Th2 response (31, 50).

EPIT (epicutaneous immunotherapy) is found to be more efficacious than other therapies, with the advantage of abundant LCs in the skin. EPIT is a needle-free treatment that is self-administrable. Through this therapy, there is an increased serum IgG level, along with a declined IgE level. Furthermore, clinical studies conducted have reported local adverse effects such as local erythema and eczema, as well as pruritus and urticaria, with no serious systemic adverse events. The non-vascularized nature of epidermal cells contributes to the lower systemic side effects. In short, the EPIT is found to be efficacious, and with a safe profile (51).

#### 4.1.3 Alternative routes

##### 4.1.3.1 Subcutaneous immunotherapy

Subcutaneous immunotherapy (SCIT) involves antigen administration by subcutaneous injection, with steadily incremented doses of the allergen, under the surveillance of a physician (52). Currently, SCIT is an effective treatment for patients with asthma and allergic rhinitis. However, SCIT is not prescribed for atopic dermatitis and food allergy (53). Several attempts were made in the past to utilize SCIT in food allergies, focusing on peanut allergy, which was efficacious, but ultimately resulted in severe systemic reactions that deemed this approach unsuitable. Numerous studies are still programmed to utilize this approach with a minimal risk of anaphylaxis, focusing on alternate routes of therapy or other modifications to the allergenic extract (54).

An IgE antibody treatment with omalizumab or adjuvants has made improvements to the efficacy and safety of SCIT allergenic extracts by reducing IgE levels significantly, and diminishing systemic side effects. Aluminum salts, mainly aluminum hydroxide, have been extensively explored as adjuvants in SCIT therapy. The mechanism of immunization in SCIT is similar to other immunotherapies that comprise the activation of DCs(dendritic cells), antigen presentation, and the stimulation of Treg cells mediating a downregulating Th2 response. SCIT, in combination with alum, has exhibited a TH1 response. Alas, the safety of normal SCIT is questioned, due to the presence of local side effects and severe systemic ADRs (55, 56).

## 5 Adjuvants in AIT

The ultimate goal of current research in allergen immunotherapy is to improve the efficacy and safety of AIT. This is achieved by developing the vaccines, along with their adjuvants, to help with reducing the dose of allergen administered. An adjuvant is a pharmaceutical aid that acts by modulating the immune response against the delivered antigen. An adjuvant requires some ideal characteristics, as follows:

- Should trigger a TH1 response;
- Should be non-mutagenic, non-carcinogenic, and non-teratogenic;
- Should be free from pyrogens;
- Stable

The adjuvants act in different ways from the depot system, rendering a slow-release does that targets the APCs and that confers an immunomodulating action. Adjuvants can act as delivery systems and immunomodulating agents (36).

Some of the adjuvants used in AIT are described below.

Alum is a first-line adjuvant that is widely used in AIT, and it is known for its fascinating property of immunomodulation and stability, along with depot formation. The antigens are adsorbed onto the alum surface, mostly by electrostatic interactions that are slowly released into the tissue and lymphatic organs. Then, the APCs take up the released antigens to process them, before presenting them on the APC surface, which would trigger the immune response (57). Three main mechanisms of alum as an adjuvant in AIT are established through studies that have been conducted.

i. Immunomodulating action through depot formation;

ii. Allergen-specific antibody production by NLRP3 inflammasome activation;

iii. Self-DNA or uric acid release triggers the activation of immature dendritic cells.

Alum, however, has an issue of non-biodegradability; therefore, research regarding its safety and toxicity must be highlighted (58).

Calcium phosphate is not widely utilized as an adjuvant in AIT (59). It is a biodegradable and biocompatible adjuvant that acts through immunomodulation via depot formation. Studies have shown that it is able to induce high IgG levels and diminish IgE levels (58).

Microcrystalline tyrosine (MCT) acts as an adjuvant in AIT, and exhibits an excellent absorption capacity, due to its biodegradable and biocompatible nature. MCT also forms a depot system that can trigger an immune response(58, 60).

Monophosphoryl lipid A (MPL) stimulates the immune response by activating the APCs, thereby inducing a TH1 response. It also activates monocytes and macrophages, fastening the process of antigen presentation. It is found to be less toxic, but due to its poor availability, it is often combined with other adjuvants to enhance its efficacy(58, 61).

## 6 Nanoparticles: A promising platform in AIT

**Figure 3:**
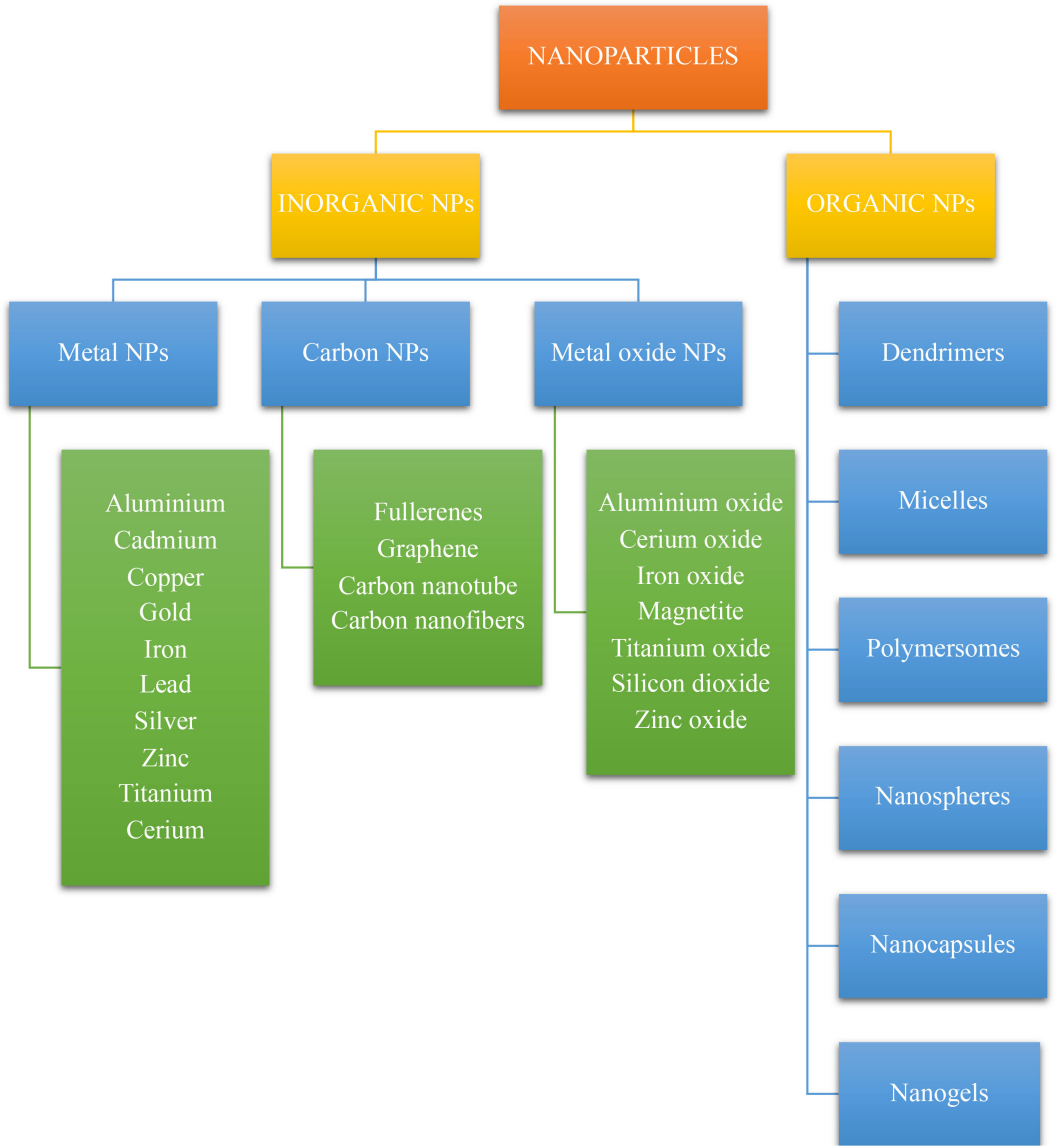
Classification of nanoparticles.

**Figure 4:**
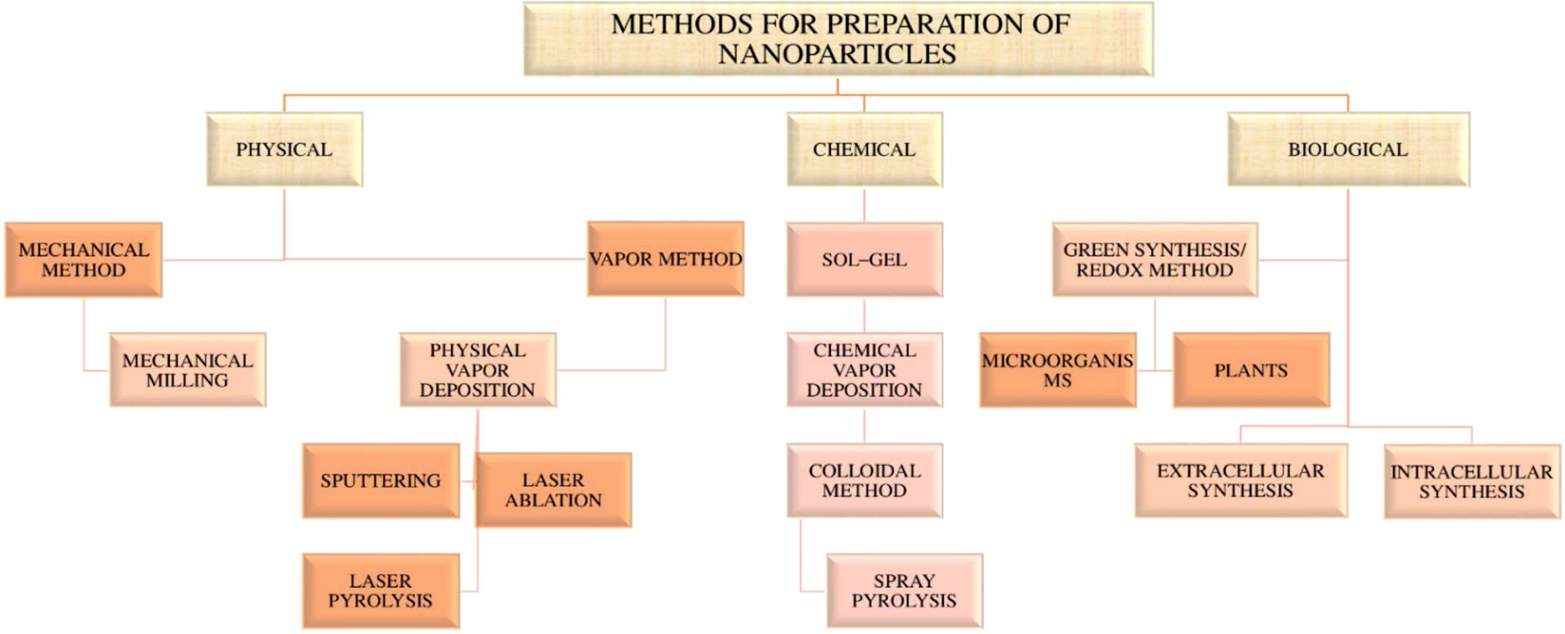
Flow chart of preparation of nanoparticles.

Nanoparticles (NPs) are defined as nano-scaled particles with a dimension of 1–100 nm which are greatly explored in drug delivery systems. Depending on the components used in synthesis and their structure, nanoparticles can be categorized as organic and inorganic nanoparticles (53, 62). The detailed classification of nanoparticles and its preparation is given in the **Figures 3** , **4** below.

Even though the conventional AIT has been found to be effective in clinical trials, serious systemic side effects are presented that have limited their application. Nanoparticles have the potential to be a promising platform in AIT, by encapsulating the allergens. NPs offer advantages as both carrier systems and adjuvants. Encapsulation benefits the allergen with triple functions, namely protection from enzymatic or acidic degradation, and delivery and co-delivery, resulting in targeted drug delivery with a reduced dose of antigen. This in turn would enhance the local drug concentration that has escaped from the immune system. The side effects of AIT can be limited, or to a certain extent, even prevented, by utilizing the peculiar feature of nanoparticle shielding of allergens which would restrict their identification through IgE that is present on the immune cells. NPs can be modified to meet the specific requirements for various routes of administration; for example, protection from gastric acids in oral delivery. Besides, drug tolerance could be improved with the targeted drug delivery of nanoparticles (63–66).

**Figure 5:**
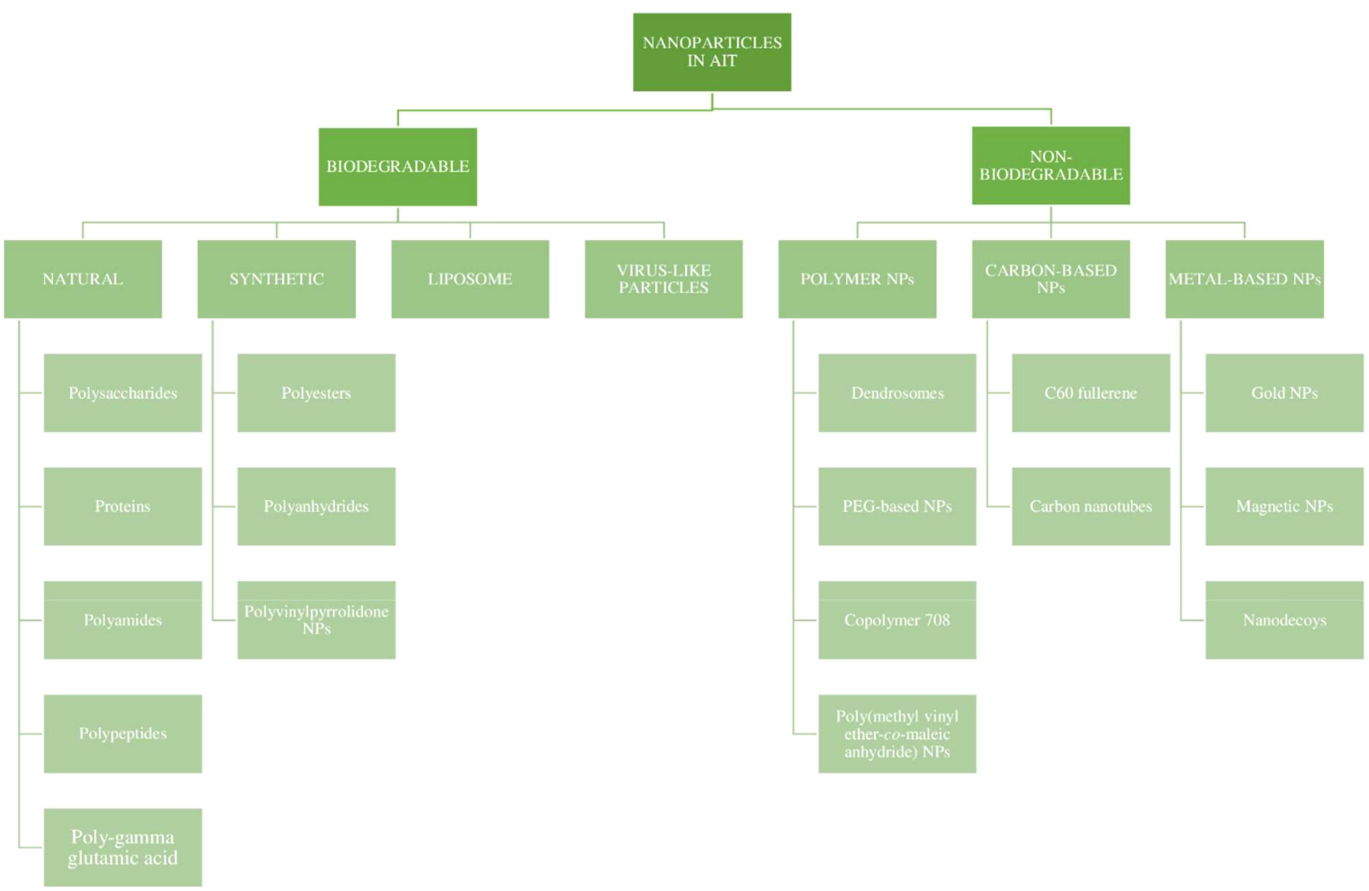
Types of nanoparticles in AIT.

The physicochemical characteristics of NPs and the attachment of specific ligands for specific targeting have greatly influenced their adjuvant effects. The encapsulation efficiency and the release pattern of a drug from a polymeric nanoparticle depends on the solid-state solubility, which is defined as the miscibility of the drug with the carrier vehicle solution (67). The particle size could affect its penetration into tissues, as well as its entry into blood vessels and the lymphatic system. The potential for antigen protection at the site of administration, and the maintenance of the stability and exertion of depot effect are attributed to the chemical nature, shape, and solubility of the NPs. The NPs also exert a direct immunosuppressive effect on the immune cells, with a prolonged circulation time. The NPs are currently being investigated for their immunomodulating effect, which could open doors to new allergen formulations in AIT (68). An attempt was made by several scientists to classify nanoparticles for the preparation of the allergen complex. Nanoparticles used in AIT can be broadly categorized as being biodegradable and non-biodegradable NPs, as represented in **Figure 5** (56, 68).

### 6.1 Biodegradable nanoparticles

**Figure 6:**
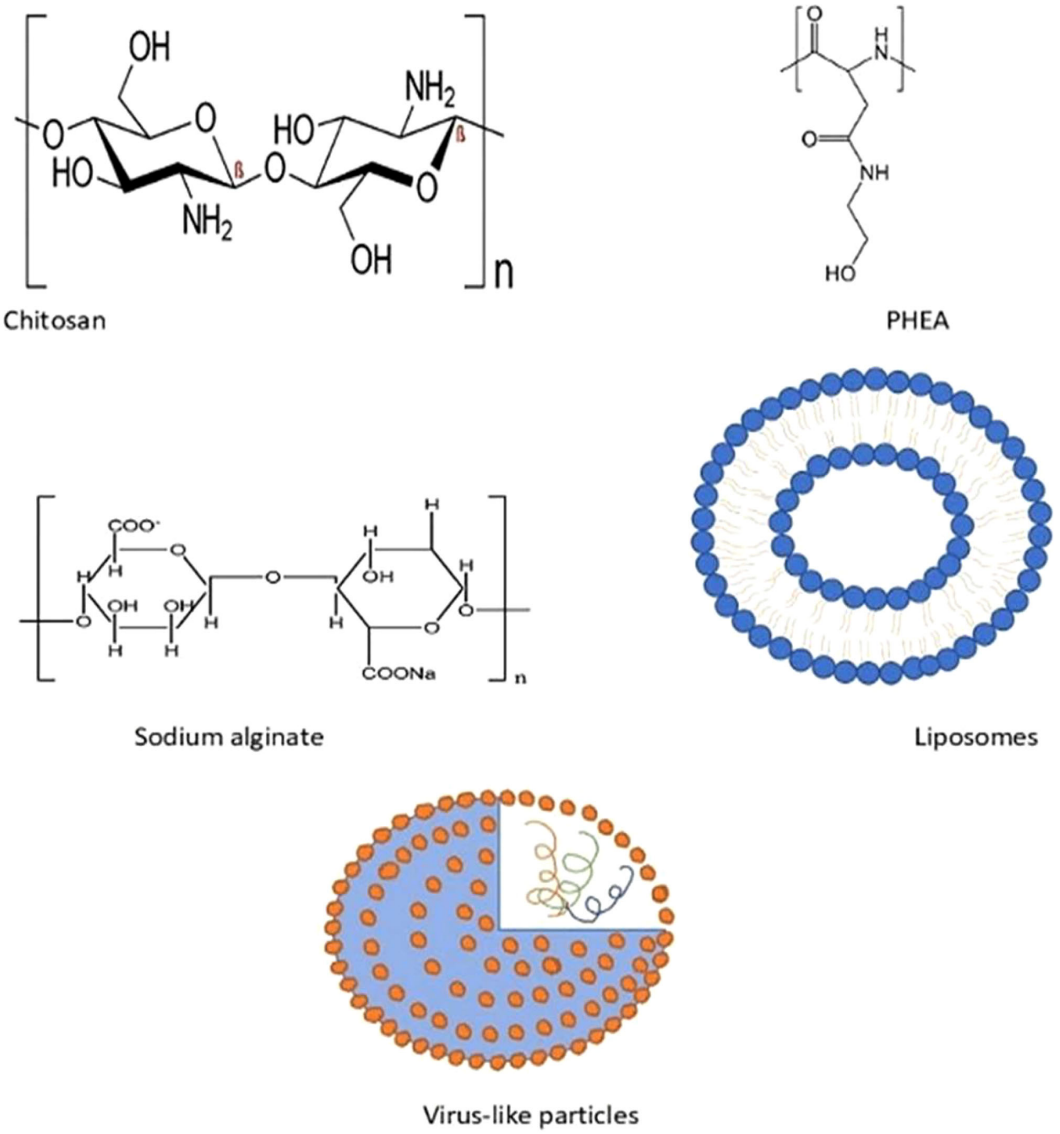
Some of the biodegradable nanostructures in AIT.

Over the past few years, various polymers have been used in formulating nanoparticles. Biodegradable nanoparticles are one of the polymers exploited intensely in the field of drug delivery systems. These nanoparticles offer numerous advantages, such as sustained/controlled release of the drug, biocompatibility, and small size; and they act as a carrier for bioactive molecules, such as proteins and peptides. Biodegradable nanoparticles comprise natural polymers (polysaccharides, proteins, polypeptides, polygammaglutamic acid, and polyamides), synthetic polymers (polyesters, polyanhydrides, and polyvinylpyrrolidones), liposomes, and virus-like particles (36, 69), as shown in **Figure 6**.

#### 6.1.1 Natural polymers

Natural polymers mainly consist of polysaccharides and polypeptides. Meanwhile, chitosan, alginate, dextran, and cellulose derivatives are some of the polysaccharide polymers. Natural polymers also comprise proteins such as collagen, gelatin, globulin, and albumin (70).

##### 6.1.1.1 Synthetic polyamides

Polyhydroxyethylaspartamide (PHEA) is one of the synthetic polypeptides that is widely utilized in drug delivery, due to its biodegradable nature and aqueous solubility. Protamine, another polyamide, has been extensively used in AIT trials. A study with protamine-based nanoparticles that are complexed with Ara h2 for peanut allergy have exhibited increased IgG antibody and diminished IgE antibody levels, and a lower reactivity upon SC administration in mice (71). Poly(gamma-glutamic acid) (ƴ, PGA) is a polypeptide, with dual functions as a carrier and an adjuvant, that has demonstrated efficacy in an investigation of ƴ-PGA with OVA, upon IV, SC, or IP administration in mice (72).

##### 6.1.1.2 Polysaccharides

Chitosan, a cationic polymer (linear polysaccharide) is extensively used in formulating nanoparticles that can maintain the immunogenicity of an allergen, along with having its own advantages of biocompatibility, biodegradability, and non-toxicity. Past studies had indicated its success in AIT in murine models with OVA and peanut, in which it had induced a Th1 response. Gelatin, a protein derived from animals, has shown a decrease in allergic symptoms when formulated as NPs. Other than that, sodium alginate allergens have exhibited a significant increase in IgG1 and IgG4 levels, along with diminished IgE levels, during immunotherapy in clinical trials.

##### 6.1.1.3 Polypeptides

Peptides derived from allergens are the most promising approaches for AIT. Based on the allergen’s basic structure, this novel treatment employs soluble synthetic allergen fragments of various lengths (73). Peptide-based vaccinations can be categorized into those that employ T-cell peptides, and those that use IgE-mediated peptides, depending on the length of the fragments and their capacity to induce tolerance (74, 75). Peptides have the ability to control the genes and activate the pathways involved in the tolerogenic response, particularly in the specific case of olive pollen. Through this, the combination of five short Ole e 1 that are derived from dodecapeptides would be able to stop the proliferative response to olive pollen associated with the release of IL-10 and IL-35, thus regulating the cytokines in allergic individuals. Additionally, these peptide combinations are incapable of activating basophils, a requirement for the creation of a novel peptide vaccine (76).

The mechanism of polypeptides was investigated in clinical trials using a mixture of peptides from Fel D 1 and from bee venom allergen. According to these studies, changes in both cellular and humoral immunity were observed. Moreover, these peptides have their own limitations, as B-cell epitopes on short peptides are weak in humoral immunotherapy (77, 78).

Another study that centralized on the usage of peptides in skin has shown that there is a significant increase in the number of Th1 cells (CD4^+^/IFN-γ^+^) and CD25^+^ cells, suggesting that a combination of immune deviation (Th2 to Th1) and regulation (the recruitment of regulatory T cells) may be important in controlling the responses to allergen post-therapy (79). There are several other studies indicating that peptides in AIT have produced satisfactory results, in which peptide-induced tolerance was observed after intradermal administration, and not observed during inhalation (80).

##### 6.1.1.4 Poly-gamma glutamic acid

Certain Bacillus strains produce high molecular weight polypeptides known as poly(gamma-glutamic acid) (or -PGA), made up of linked glutamic acid units and carboxylate side chains. The amphiphilic characteristics of the hydrophobically modified -PGA copolymers makes it possible to create nanoparticles through a straightforward process (81, 82). Further research has revealed that these particles could activate human monocyte-derived dendritic cells, thus significantly increasing the production of chemokines and inflammatory cytokines, as well as costimulatory molecules and immunomodulatory mediators that are essential for effective T-cell priming. Additionally, *in vitro* research using grass pollen allergen Phleum pratense-loaded -PGA nanoparticles and monocyte-derived dendritic cells has revealed an increase in allergen-specific IL-10 production and the proliferation of autologous CD4+ memory T cells. As for allergen-specific immunotherapy, these systems appear to be innovative and effective adjuvants and antigen carriers (82).

A study by Touseefn et al. has revealed that (POSS)-grafted polyurethane (PU) is a good nanomaterial for muscle tissue renewal. Thus, it can be inferred that this nanomaterial is a good material for immunotherapy (83). Another study by the same author outlines that a HAP (hydroxyapatite scaffold) with N. sativa grafts can be combined for use as implants. Interestingly, N. sativa-grafted HAP has demonstrated a remarkable potential as effective antiosteoporotic scaffolds, inheriting antioxidant and anti-inflammatory properties; as a result, they can be used as forthcoming material for allergic reactions (84).

#### 6.1.2 Synthetic polymers

*Polyesters*: Poly(lactic-co-glycolide) (PLGA) and polylactic acids have been extensively researched in drug delivery systems, due to their biodegradability and biocompatibility nature (85). These polymers have been subjected to extensive studies in AIT to identify their potential to specifically target antigen-presenting cells (APCs). The acidic degradation products might negatively affect the stability of the loaded antigen, which can be addressed by the use of copolymers such as PEG. Several studies have shown that the application of PLGA-based NPs has superior effects on free antigens. The oral administration of PLGA-based NPs in mice has displayed a Th1 immune response with an elevated IgG level, and diminished IgE levels, while other studies have shown a conversion from Th2 to Th1 immune response (86). Poly(ϵ-caprolactone) (PCL) is another polymer that is biocompatible, biodegradable, and semicrystalline. The *in vivo* degradation of PCL is much slower than PLGA; therefore, it can be used for the prolonged delivery of drugs/antigens. Treatment with OVA-PCL in mice had evoked an enhanced IgG level and lowered IgE levels, which resulted in declined allergic symptoms.

##### 6.1.2.1 Polyanhydrides

The stability of encapsulated antigen is maintained with polyanhydride, due to its non-toxic and reduced acidic degradation products, which make them more suitable for antigen delivery than polyesters. The bio-adhesive properties of these polymers would contribute to the Th1 response, due to its enhanced interaction between the antigen and APCs in the mucosa. An enhanced Th1 response and Treg cytokines were observed with spray-dried peanut protein-loaded NP immunization in mice. Oral immunization with polyanhydride NPs containing cashew nut proteins (single dose) led to the induction of Th1 and Treg immune responses, exhibiting their immunomodulatory properties, as seen in a study conducted by Pereira et al. (87).

##### 6.1.2.2 Polyvinylpyrrolidones

There are several studies that imply an interest in employing polyvinylpyrrolidone (PVP) to generate promising allergen delivery nanocarriers, despite having less workers than the polymers discussed previously (36). For instance, Aspergillus fumigatus was enclosed in poly(vinylpyrrolidone) nanoparticles by Madan et al., who were able to exhibit intact integrity, immunoreactivity, and persistent allergen release in just 9 weeks (88).

In addition to these polymers, a study by Touseef et al. has shown that polyvinyl alcohol can be used to encapsulate L. gasseri, which possesses extensive usage in food technology. Based on this study, it can be implied that the nanomaterial polyvinyl alcohol with modification can be used for the management of food allergies, by improving its stability (89).

#### 6.1.3 Liposomes

Liposomes are small spherical vesicular structures comprising phospholipid bilayer that encapsulate hydrophilic drugs, thus insulating them from the aqueous environment. Liposomes have been continuously explored for drug delivery and their adjuvant effects, as they can enhance the bioavailability and solubility of the targeted drug, as well as optimizing specific targeting at the site of action without unwanted side effects. It has been proven that liposomes are able to elicit immunomodulatory functions. Several studies with allergen-loaded liposomes ended up with a conclusion that they were effective in inducing a Th1 immune response and an increase in Treg cytokines. These liposomes were found to be safe and effective in AIT, with pollen grains, dust mites, and cat allergen, which resulted in declined serum IgE levels. However, human clinical trials of AIT with allergen-loaded liposomes concluded that liposomes were not suitable for AIT, due to its systemic safety issues. The modified liposomes triggered CD8+ Treg, which subdued the allergic symptoms in the murine food allergy model. Contrary to that, liposomes loaded with refined protein Per a 9 were found to be effective in mitigating allergic symptoms. Aliu et al. (90) investigated the efficacy of liposomal delivery of allergen through SLIT, and concluded that prophylactic SLIT with OVA liposomes were more effective in excluding allergic symptoms than the free OVA AIT (91). Nevertheless, systemic safety in humans must be addressed, to enhance the effectiveness of liposomes in AIT (69).

#### 6.1.4 Virus-like particles

Virus-like particles (VLPs) are self-assembling and non-infectious nanoparticles that have been utilized in drug delivery systems or as adjuvants. In a randomized clinical trial, treatment with VLPs among house dust mite-allergic patients helped with alleviating allergic symptoms by mediating a Th1 immune response. The SC administration of Fel d 1 in mice was found to be efficacious, without triggering any systemic side effects (92). Adeno-associated VLP, when administered in mice, elicited heightened IgG levels and diminished allergen-specific IgE without inducing any anaphylactic reactions. Thus, VLPs can be considered as an option to enhance the efficacy of AIT.

### 6.2 Non-biodegradable nanoparticles

Researches have been conducted on non-biodegradable NPs, which are made up of different materials, such as gold, silica, and polymers, for immunization in antigen delivery, by presenting them to the immune system for an extended period. Polymer NPs were found to be effective against the OVA peptide, eliciting a shift to the Th1 response. The conjugation of OVA with a copolymer of N-vinylpyrrolidone and maleic anhydride in AIT resulted in a remarkable decline of IgE levels, with an enhanced IgG level. Polyethylene glycol (PEG) is a water-soluble, non-biodegradable polymer that has been explored extensively in drug delivery. In human trials, the encapsulation of allergens with PEG NPs led to a targeted allergen delivery, and triggered the Th1 immune response, which further proved their potential as adjuvants in AIT. However, the safety profile of these NPs is still under research, despite its biocompatible nature. Further research must be conducted to fulfill the needs of these NPs, particularly its degradation in the body, as the non-clearance of these carriers may end up with unwanted side effects (69).

#### 6.2.1 Polymer nanoparticles

Dendrimers, which are branched polymeric nanoparticles, were found to have a role in decreasing IgE, along with a decreased Th1 response (93). In another pre-clinical study by Garaczi et al., the topical administration of nanoparticles, by mixing with poly(ethylene imine) with OVA pDNA, reduced nasal symptoms of rhinitis and induced balanced Th1/Th2 responses. When the copolymers of ethylene oxide and propylene oxide (poloxamine) nanospheres were examined for their therapeutic effects on asthmatic mice, a drop in inflammatory cytokines in the BAL, and a reduction in airway hyperreactivity were observed. PEG, an aliphatic polyether that is used in medicinal applications, is the most well-known synthetic water-soluble polymer. Due to its distinct characteristics, PEG is also known as the “gold standard” for biomedical applications (94). PEG is a non-biodegradable polymer; hence, cleavage sites must be included for the regulated delivery of proteins by PEG-based NP in AIT to take place. In order to achieve this, Pohlit et al. created acid-labile PEG macromonomers that would break down at pH 5 (the physiological pH inside the endolysosome). During *in vitro* tests in humans, the encapsulation of allergens into these NPs led to targeted administration, the activation of T-cell proliferation, and allergen shielding from identification by IgE antibodies (95). A study by Maria et al. shows as a sublingual immunotherapy glycosylated nanostructures can induce a long lasting tolerance in lipid transfer proteins (LTP) induced allergey.LTP allergy is mainly generated from fruits such as peach and apple (96).

#### 6.2.2 Carbon-based nanoparticles

Carbon tubes are the most commonly investigated nanoparticles for drug targeting (97). A study on the immunomodulatory activity of these carbon tubes revealed that they can either promote or suppress the immune response, and they can be used as an appropriate adjuvant. It is also important to highlight that the negative or toxic effects of these nanotubes were also reported (98). Another study by Ryan et al. also demonstrated that C 60 fullerene had an inhibitory effect on IgE-mediated release from mast cells and basophils. Furthermore, it had the ability to block signaling molecules and cytoplasmic ROS, which prevented the release of histamine and anaphylaxis (99). In multiple pre-clinical studies, the administration of tetraglycolate fullerenes or a water-soluble form of C60 fullerene to OVA-sensitized mice caused a shift in cytokine production from Th2 to Th1, as well as a reduction in airway inflammation, eosinophilia, and bronchoconstriction, which were brought on by the production of an anti-inflammatory P-450 eicosanoid metabolite or an increase in Foxop 3 and fillagrin m RNA (100, 101).

#### 6.3.3 Metal-based nanoparticles

Gold nanoparticles have been one of the most commonly used drug delivery systems for anti-inflammatory and antioxidant effects (102).Through magnetic NPs coated in dextran and coupled to either bovine b-lactoglobulin or ovomucoid, Marengo et al. proposed a potential delivery platform for AIT, as it was observed, through the utilization of confocal laser scanning microscopy to confirm internalization, that human monocytes absorbed conjugated NPs more readily than nonconjugated NPs (103, 104).

The reported studies of nanoparticles for allergen mediated immunotherapy is given in **Table 2** below.

**Table 2:** Reported studies of nanoparticles for allergen mediated immunotherapy.

Allergen source	Nanoparticle component	Species	Findings of studies	Reference
Ara h 2 extracted from raw peanuts	Protamine-based nanoparticles (proticles) with CpG-oligodeoxynucleotides	BALB/c mice	Because they decrease the Th2-dominated immune response that is triggered by an allergen, biodegradable nanoparticles based on protamine and containing CpG-ODN are a new carrier system that can be used for allergy immunotherapy.	(71)
Raw or roasted peanuts	Poly(anhydride) nanoparticles	C57BL/6 mice	A pro-TH1 immune response was induced as a result of oral immunization with poly(anhydride) NPs, particularly those formulations that were spray-dried.	(105)
Peanut allergen gene (pCMVArah2)	Chitosan–DNA nanoparticles	AKR/J mice	Oral allergen immunization conating chitosan-DNA nanoparticle is very effective in anaplylatic reaction and can be used for prophylactic management.	(106)
Roasted peanut extract	Polymer conjugate of mannosamine to the copolymer of methyl vinyl ether and maleic anhydride	CD1 mice	Evidence from previous studies suggests that pretreatment with BLG-Pep+CpG/NP modifies the DC phenotype, suppresses the formation of Th2 cells, and enhances the activity of regulatory T cells and helper T cells, all of which may assist to inhibit the growth of allergen-specific CMA.	(107)
Peanut protein allergen Ara h 2	Poly(lactide-co-glycolide acid) (PLGA) nanoparticle	Liver sinusoidal endothelial cells (LSECs)	The targeted distribution of carefully selected T-cell epitopes to naturally tolerogenic liver APC was shown to have the potential to build an effective therapy platform for peanut allergy anaphylaxis	(86)
Wheat allergen in milk	Gold nanoparticles		Gold nanoparticle-based lateral flow (LFIA) strips rapidly detected gliadin in negative milk with a visible limit of detection of 25 ng/mL and a computed LOD value of 6.56 ng/mL. Positive samples tested with LFIA strips yielded responses that were significantly consistent with sandwich enzyme-linked immunosorbent results. Consequently, the LFIA strip rapidly and accurately detects the wheat allergen in milk.	(108)
Cows milk allegen	PLGA nanoparticles	mice	This study reported that oral administration of PLGA nanoparticle encapsulate beta lactoglobulin peptide can prevent rise in serum BLG specific IgE	(109)

## 7 Future prospects

Allergen mediated immunotherapy is novel therapeutic form established for the management of the common allergen sources. Allergen immunotherapy, which act by repeat administration of allergen extracts and can offer a permanent solution for allergic reactions. Recently administration through oral especially sub-lingual route has emerged safe and effective.

The discovery of molecular allergology has made it feasible to create recombinant allergens, which has led to greater accuracy in allergy diagnosis as well as in the selection of patients for allergen immunotherapy. In regards to efficacy or safety, recombinant allergen immunotherapy does not now provide any benefits over entire allergen extracts that are currently accessible; however, in the future, recombinant hypoallergenic variants might provide such advantages. Personalized vaccinations containing significant allergens or hypoallergenic modifications may one day be employed in immunotherapy. These injections would be tailored to patients’ individual IgE sensitivity profiles.

Ongoing research into the prevention of other food allergies, such as those to prawns, cashews, and milk, is being conducted on infants who are considered to be at risk for developing those allergies. This is because early introduction of peanut as a primary prophylactic method has been shown to be extraordinarily effective while also being completely risk-free. As a result, it makes perfect sense to contemplate the primary preventative measures for allergic reactions.

The application of nanotechnology in immunotherapy for the treatment of food allergies is a relatively new platform. It contributes to the development of a new treatment approach for improving the clinical safety and effectiveness of immunotherapy. The use of nanoparticles in AIT is being ramped up as a result of the results obtained thus far. The impact that NP has on the immune system is relevant to allergen immunotherapy, which is a form of treatment for allergic reactions. For this reason, more research is required to show the positive effects of various delivery methods and adjuvant formulations in human studies.

## 8 Conclusion

Food allergies are becoming more common, with increasing severity, which have sparked a great interest to discover commercial treatment strategies. Nanoparticles, DNA vaccines, adjuvants, and combination therapies are some of the methods being investigated to reduce the negative effects of AIT, as well as to increase the effectiveness and development of permanent oral tolerance. Native and modified allergens can be produced by using recombinant technology with less allergenicity. Future research into hypoallergenic IT agents may be particularly significant, due to rising concerns regarding the clinical safety of food allergy therapy methods.

The pervasiveness of food-induced anaphylaxis has led to research in AIT, which is an impressive and powerful tool in mitigating allergic symptoms. Diversified routes of administration and delivery systems would be able to enhance the effectiveness of AIT. Although the efficacy of AIT was established through numerous studies, its safety profile should be examined continuously to further prove its advantages over other therapies. Intralymphatic and intranasal routes of allergen delivery can be utilized to improve effectiveness. The targeted drug delivery of nanoparticles into the lymphatic system is much more efficacious than conventional delivery system, as it can bypass the systemic side effects. Nanoparticles have displayed their effectiveness in allergen immunotherapy, but the toxicity assessment must be highlighted, to ensure the safety profile. Abundant AIT trials have been conducted, but attentive studies must be conducted to fulfill the required expectations of safety and systemic side effects. AIT is the core component in the management of allergic diseases; therefore, its advantages should be established. AIT with versatile routes of administration such as SLIT, and delivery systems such as nanoparticles, could open many doors for more promising platforms in the prevention of food-induced anaphylaxis.

In the context of trials associated with AIT, establishing the baseline outcome measurement that can be used to predict the likelihood of a successful AIT, and to track the immunological response to intervention during the course of the AIT, both in the short, and long term, remain as a challenge. These goals can be accomplished by standardizing the outcome metrics used across each clinical study.

Even though the usage of nanotechnology has grown in popularity in recent years for immunization procedures, allergen immunotherapy is still in its infancy stage. Despite the positive outcomes of *in vivo* nanoparticle investigations, a deeper comprehension of nanoparticle–allergen complexes and their molecular interactions with the immune system is necessary. Investigating the effect of the nanoparticle–allergen complex at each mechanistic step underlying the immune response, such as internalization, maturation, antigen processing, presentation, and the activation of T cell differentiation, can lead to the development of safe and effective adjuvants for AIT. Therefore, more research on the immunomodulatory effects of polymeric NPs is essential to expand our knowledge, and consequently, our capacity to design more precise and efficient allergen-specific immunotherapies.

## Author contributions

Conceptualization, VV and SK. Data collection, VV, SK and AT. Formal analysis, AT, SF and SK. Writing—original draft preparation, SK, AT, RS, and VV. Writing—review and editing, JK, RS, SAF, SM and MN. Supervision, VV. All authors contributed to the article and approved the submitted version.

## References

[1] Hiera F Viola I Spinuzza A Caminiti L Crisafulli G Panasiti I. Allergen-specific immunotherapy for im-munoglobulin E-mediated food allergy. Eur Med J (2019) 4:95–100. doi: 10.33590/emj/10310420

[2] Saritha CK Jose J Viswanad V. Allergen Immunotherapy: Tactic in manipulating food allergen induced anaphylaxis. Int J Res Pharm Sci (2020) 11:2533–42. doi: 10.26452/ijrps.v11i2.2250

[3] Akdis CA Akdis M. Mechanisms of allergen-specific immunotherapy and immune tolerance to allergens. World Allergy Organ J (2015) 8:17. doi: 10.1186/s40413-015-0063-2 26023323 PMC4430874

[4] Campuzano S Ruiz-Valdepeñas Montiel V Serafín V Yáñez-Sedeño P Pingarrón JM. Cutting-edge advances in electrochemical affinity biosensing at different molecular level of emerging food allergens and adulterants. Biosensors (Basel) (2020) 10:10. doi: 10.3390/bios10020010 32041251 PMC7168206

[5] Yu W Freeland DMH Nadeau KC. Food allergy: immune mechanisms, diagnosis and immunotherapy. Nat Rev Immunol (2016) 16:751–65. doi: 10.1038/nri.2016.111 PMC5123910 27795547

[6] Pajno GB Fernandez-Rivas M Arasi S Roberts G Akdis CA Alvaro-Lozano M. EAACI Guidelines on allergen immunotherapy: IgE-mediated food allergy. Allergy (2018) 73:799–815. doi: 10.1111/all.13319 29205393

[7] Aquino A Conte-Junior CA. A systematic review of food allergy: Nanobiosensor and food allergen detection. Biosensors (Basel) (2020) 10:194. doi: 10.3390/bios10120194 33260424 PMC7760337

[8] Gómez-Arribas L Benito-Peña E Hurtado-Sánchez M Moreno-Bondi M. Biosensing based on nanoparticles for food allergens detection. Sensors (Basel) (2018) 18:1087. doi: 10.3390/s18041087 29617319 PMC5948517

[9] Rai M Ingle AP Yadav A Golińska P Trzcińska-Wencel J Rathod S. Nanotechnology as a promising approach for detection, diagnosis and treatment of food allergens. Curr Nanosci (2023) 19:90–102. doi: 10.2174/1573413718666220426101432

[10] Burton OT Noval Rivas M Zhou JS Logsdon SL Darling AR Koleoglou KJ. Immunoglobulin E signal inhibition during allergen ingestion leads to reversal of established food allergy and induction of regulatory T cells. Immunity (2014) 41:141–51. doi: 10.1016/j.immuni.2014.05.017 PMC4123130 25017467

[11] Vighi G Marcucci F Sensi L Di Cara G Frati F. Allergy and the gastrointestinal system. Clin Exp Immunol (2008) 153:3–6. doi: 10.1111/j.1365-2249.2008.03713.x 18721321 PMC2515351

[12] Anvari S Miller J Yeh C-Y Davis CM. IgE-mediated food allergy. Clin Rev Allergy Immunol (2019) 57:244–60. doi: 10.1007/s12016-018-8710-3 30370459

[13] Spergel JM. Nonimmunoglobulin E-mediated immune reactions to foods. Allergy Asthma Clin Immunol (2006) 2:78–85. doi: 10.1186/1710-1492-2-2-78 PMC2876187 20525161

[14] Calvani M Anania C Cuomo B D’Auria E Decimo F Indirli GC. Non–IgE, or mixed IgE/non–IgE-mediated gastrointestinal food allergies in the first years of life: Old and new tools for diagnosis. Nutrients (2021) 13:226. doi: 10.3390/nu13010226 33466746 PMC7829867

[15] Al-Farga A. Food allergy, classification, symptoms, diagnosis and prevention-review. Int J Agric Res (2017) 4(1):1–5.

[16] Caio G. Non-IgE/mixed food allergies and functional gastrointestinal disorder: A common thread between childhood and adulthood. Nutrients (2022) 14:835. doi: 10.3390/nu14040835 35215484 PMC8879813

[17] Cianferoni A. Non-IgE mediated food allergy. Curr Pediatr Rev (2020) 16:95–105. doi: 10.2174/1573396315666191031103714 31670623

[18] Moran TP Vickery BP Burks AW. Oral and sublingual immunotherapy for food allergy: current progress and future directions. Curr Opin Immunol (2013) 25:781–7. doi: 10.1016/j.coi.2013.07.011 PMC3935613 23972904

[19] Bartemes KR Kita H. Roles of innate lymphoid cells (ILCs) in allergic diseases: The 10-year anniversary for ILC2s. J Allergy Clin Immunol (2021) 147:1531–47. doi: 10.1016/j.jaci.2021.03.015 PMC8114584 33965091

[20] Sahiner UM Layhadi JA Golebski K István Komlósi Z Peng Y Sekerel B. Innate lymphoid cells: The missing part of a puzzle in food allergy. Allergy (2021) 76:2002–16. doi: 10.1111/all.14776 33583026

[21] Schuijs MJ Png S Richard AC Tsyben A Hamm G Stockis J. ILC2-driven innate immune checkpoint mechanism antagonizes NK cell antimetastatic function in the lung. Nat Immunol (2020) 21:998–1009. doi: 10.1038/s41590-020-0745-y 32747815 PMC7116357

[22] Jin J Sunusi S Lu H. Group 2 innate lymphoid cells (ILC2s) are important in typical type 2 immune-mediated diseases and an essential therapeutic target. J Int Med Res (2022) 50:3000605211053156. doi: 10.1177/03000605211053156 35048721 PMC8796086

[23] Roan F Obata-Ninomiya K Ziegler SF. Epithelial cell–derived cytokines: more than just signaling the alarm. J Clin Invest (2019) 129:1441–51. doi: 10.1172/jci124606 PMC6436879 30932910

[24] Mayorga C Palomares F Cañas JA Pérez-Sánchez N Núñez R Torres MJ. New insights in therapy for food allergy. Foods (2021) 10:244–60. doi: 10.3390/foods10051037 PMC8151532 34068667

[25] Nurmatov U Dhami S Arasi S Pajno GB Fernandez-Rivas M Muraro A. Allergen immunotherapy for IgE-mediated food allergy: a systematic review and meta-analysis. Allergy (2017) 72:1133–47. doi: 10.1111/all.13124 28058751

[26] Yang X Liang R Xing Q Ma X. Fighting food allergy by inducing oral tolerance: Facts and fiction. Int Arch Allergy Immunol (2021) 182:852–62. doi: 10.1159/000515292 33895737

[27] Dillon A Lo DD. M cells: Intelligent engineering of mucosal immune surveillance. Front Immunol (2019) 10:1499. doi: 10.3389/fimmu.2019.01499 31312204 PMC6614372

[28] Stagg AJ. Intestinal dendritic cells in health and gut inflammation. Front Immunol (2018) 9:2883. doi: 10.3389/fimmu.2018.02883 30574151 PMC6291504

[29] Tham EH Rajakulendran M Lee BW Van Bever HPS. Epicutaneous sensitization to food allergens in atopic dermatitis: What do we know? Pediatr Allergy Immunol (2020) 31:7–18. doi: 10.1111/pai.13127 31541586

[30] Sampson HA O’Mahony L Burks AW Plaut M Lack G Akdis CA. Mechanisms of food allergy. J Allergy Clin Immunol (2018) 141:11–9. doi: 10.1016/j.jaci.2017.11.005 29307410

[31] Senti G von Moos S Kündig TM. Epicutaneous allergen administration: is this the future of allergen-specific immunotherapy?: Epicutaneous allergen immunotherapy. Allergy (2011) 66:798–809. doi: 10.1111/j.1398-9995.2011.02560.x 21518374

[32] Nowak-Węgrzyn A Sampson HA. Future therapies for food allergies. J Allergy Clin Immunol (2011) 127:558–73. doi: 10.1016/j.jaci.2010.12.1098 PMC3066474 21277625

[33] Gernez Y Nowak-Węgrzyn A. Immunotherapy for food allergy: Are we there yet? J Allergy Clin Immunol Pract (2017) 5:250–72. doi: 10.1016/j.jaip.2016.12.004 28283151

[34] Schmidt A Oberle N Krammer PH. Molecular mechanisms of treg-mediated T cell suppression. Front Immunol (2012) 3:51. doi: 10.3389/fimmu.2012.00051 22566933 PMC3341960

[35] Nair N Vinod V Suresh MK Vijayrajratnam S Biswas L Peethambaran R. Amidase, a cell wall hydrolase, elicits protective immunity against Staphylococcus aureus and S. epidermidis. Int J Biol Macromol (2015) 77:314–21. doi: 10.1016/j.ijbiomac.2015.03.047 25841371

[36] De Souza Rebouças J Esparza I Ferrer M Sanz ML Irache JM Gamazo C. Nanoparticulate adjuvants and delivery systems for allergen immunotherapy. J Biomed Biotechnol (2012) 2012:1–13. doi: 10.1155/2012/474605 22496608 PMC3303624

[37] Mori F Barni S Liccioli G Novembre E. Oral Immunotherapy (OIT): A personalized medicine. Medicina (Kaunas) (2019) 55:684. doi: 10.3390/medicina55100684 31614929 PMC6843277

[38] Burks; SH Robyn W Bacharier; DB Khurana OL Hershey Gurjit, Stokes R. Middleton’s allergy : principles and practice. Amsterdam, The Netherlands: Elsevier (2020).

[39] Santos AF James LK Bahnson HT Shamji MH Couto-Francisco NC Islam S. IgG4 inhibits peanut-induced basophil and mast cell activation in peanut-tolerant children sensitized to peanut major allergens. J Allergy Clin Immunol (2015) 135:1249–56. doi: 10.1016/j.jaci.2015.01.012 PMC4418748 25670011

[40] Kanagaratham C El Ansari YS Lewis OL Oettgen HC. IgE and IgG antibodies as regulators of mast cell and basophil functions in food allergy. Front Immunol (2020) 11:603050. doi: 10.3389/fimmu.2020.603050 33362785 PMC7759531

[41] Wood RA. Food allergen immunotherapy: Current status and prospects for the future. J Allergy Clin Immunol (2016) 137:973–82. doi: 10.1016/j.jaci.2016.01.001 27059725

[42] Pullanikkad AP Nambiar N. An overview on allergen immunotherapy for allergic asthma with emphasis on subcuta-neous and sublingual immunotherapy | Annals of the rOmanian society for cell biology. Overview Allergen Immunotherapy Allergic Asthma Emphasis Subcuta-neous Sublingual Immunotherapy | Ann ROmanian Soc Cell Biol (2021) 25:10571–80.

[43] Jutel M Bartkowiak-Emeryk M Bręborowicz A Cichocka-Jarosz E Emeryk A Gawlik R. Sublingual immunotherapy (SLIT) – indications, mechanism, and efficacy Position paper prepared by the Section of Immunotherapy, Polish Society of Allergy. Ann Agric Environ Med (2015) 23:44–53. doi: 10.5604/12321966.1196851 27012173

[44] Moingeon P Lombardi V Baron-Bodo V Mascarell L. Enhancing allergen-presentation platforms for sublingual immunotherapy. J Allergy Clin Immunol Pract (2017) 5:23–31. doi: 10.1016/j.jaip.2016.07.020 28065340

[45] Aswathy KN Asdaq SMB Saritha CK Thomas L Haridas N Viswanad V. Formulation and in-vitro characterization of fast-disintegrating herbal extract sublingual immunotherapy tablet for peanut-induced allergic asthma. Saudi J Biol Sci (2022) 29:1283–97. doi: 10.1016/j.sjbs.2021.12.031 PMC8913557 35280568

[46] Gomez-Casado C Sanchez-Solares J Izquierdo E Díaz-Perales A Barber D Escribese MM. Oral mucosa as a potential site for diagnosis and treatment of allergic and autoimmune diseases. Foods (2021) 10:970. doi: 10.3390/foods10050970 33925074 PMC8146604

[47] Gomez F Bogas G Gonzalez M Campo P Salas M Diaz-Perales A. The clinical and immunological effects of Pru p 3 sublingual immunotherapy on peach and peanut allergy in patients with systemic reactions. Clin Exp Allergy (2017) 47:339–50. doi: 10.1111/cea.12901 28160513

[48] Canonica GW Cox L Pawankar R Baena-Cagnani CE Blaiss M Bonini S. Sublingual immunotherapy: World Allergy Organization position paper 2013 update. World Allergy Organ. J (2014) 7:6. doi: 10.1186/1939-4551-7-6 24679069 PMC3983904

[49] Casale TB Stokes JR. Immunotherapy: what lies beyond. J Allergy Clin Immunol (2014) 133:612–9. doi: 10.1016/j.jaci.2014.01.007 24581428

[50] Weiss R Scheiblhofer S MaChado Y Thalhamer J. New approaches to transcutaneous immunotherapy: Targeting dendritic cells with novel allergen conjugates. Curr Opin Allergy Clin Immunol (2013) 13:669–76. doi: 10.1097/aci.0b013e328364f4df PMC3814987 24169433

[51] Wang J Sampson HA. Safety and efficacy of epicutaneous immunotherapy for food allergy. Pediatr Allergy Immunol (2018) 29:341–9. doi: 10.1111/pai.12869 29369411

[52] Feuille E. Nowak-wegrzyn, A. Allergen-specific immunotherapies for food allergy. Allergy Asthma Immunol Res (2018) 10:189–206. doi: 10.4168/aair.2018.10.3.189 29676066 PMC5911438

[53] Song TW. A practical view of immunotherapy for food allergy. Korean J Pediatr (2016) 59:47–53. doi: 10.3345/kjp.2016.59.2.47 26958062 PMC4781731

[54] Nelson HS. Allergen immunotherapy: Where is it now? J Allergy Clin Immunol (2007) 119:769–77. doi: 10.1016/j.jaci.2007.01.036 17337297

[55] He P Zou Y Hu Z. Advances in aluminum hydroxide-based adjuvant research and its mechanism. Hum Vaccin. Immunother (2015) 11:477–88. doi: 10.1080/21645515.2014.1004026 PMC4514166 25692535

[56] Gamazo C D’Amelio C Gastaminza G Ferrer M. and irache, J Adjuvants for allergy immunotherapeutics. M.Hum Vaccin. Immunother (2017) 13:2416–27. doi: 10.1080/21645515.2017.1348447 PMC5647973 28825867

[57] Ghimire TR. The mechanisms of action of vaccines containing aluminum adjuvants: an in *vitro* vs in *vivo* paradigm. Springerplus (2015) 4:181. doi: 10.1186/s40064-015-0972-0 25932368 PMC4406982

[58] Johnson L Duschl A Himly M. Nanotechnology-based vaccines for allergen-specific immunotherapy: Potentials and challenges of conventional and novel adjuvants under research. Vaccines (Basel) (2020) 8:237. doi: 10.3390/vaccines8020237 32443671 PMC7349961

[59] Klimek L Schmidt-Weber CB Kramer MF Skinner MA Heath MD. Clinical use of adjuvants in allergen-immunotherapy. Expert Rev Clin Immunol (2017) 13:599–610. doi: 10.1080/1744666x.2017.1292133 28162007

[60] Heath MD Swan NJ Marriott AC Silman NJ Hallis B Prevosto C. Comparison of a novel microcrystalline tyrosine adjuvant with aluminium hydroxide for enhancing vaccination against seasonal influenza. BMC Infect Dis (2017) 17:232. doi: 10.1186/s12879-017-2329-5 28347293 PMC5369220

[61] Mohsen MO Heath MD Cabral-MIranda G Lipp C Zeltins A Sande M. Vaccination with nanoparticles combined with micro-adjuvants protects against cancer. J Immunother. Cancer (2019) 7:114. doi: 10.1186/s40425-019-0587-z 31027511 PMC6485085

[62] Chenthamara D Subramaniam S Ramakrishnan SG Krishnaswamy S Essa MM Lin F-H. Therapeutic efficacy of nanoparticles and routes of administration. Biomater. Res (2019) 23:20. doi: 10.1186/s40824-019-0166-x 31832232 PMC6869321

[63] Sasikumar A Kamalasanan K. Nanomedicine for prostate cancer using nanoemulsion: A review. J Control. Release (2017) 260:111–23. doi: 10.1016/j.jconrel.2017.06.001 28583444

[64] Annu S Qamar Z Md S Alhakamy NA Baboota S Ali J. An insight to brain targeting utilizing polymeric nanoparticles: Effective treatment modalities for neurological disorders and brain tumor. Front Bioeng. Biotechnol (2022) 10:788128. doi: 10.3389/fbioe.2022.788128 35186901 PMC8851324

[65] Cadinoiu AN Rata DM Atanase LI Mihai CT Bacaita SE Popa M. Formulations based on drug loaded aptamer-conjugated liposomes as a viable strategy for the topical treatment of basal cell carcinoma-*in vitro* tests. Pharmaceutics (2021) 13:866. doi: 10.3390/pharmaceutics13060866 34208362 PMC8231244

[66] Sur S Rathore A Dave V Reddy KR Chouhan RS Sadhu V. Recent developments in functionalized polymer nanoparticles for efficient drug delivery system. Nano-struct nano-objects (2019) 20:100397. doi: 10.1016/j.nanoso.2019.100397

[67] Radhakrishnan R Kamalasanan K. Pharmaceutical perspectives of selection criteria and toxicity profiling of nanotheranostic agents. In: Drug delivery nanosystems for biomedical applications. Elsevier (2018). p. 45–74.

[68] Felice G Di Colombo P. Nanoparticle-allergen complexes for allergen immunotherapy. Int J Nanomed (2017) 12:4493–504. doi: 10.2147/IJN.S134630 PMC5484593 28684909

[69] Pohlit H Bellinghausen I Frey H Saloga J. Recent advances in the use of nanoparticles for allergen-specific immunotherapy. Allergy (2017) 72:1461–74. doi: 10.1111/all.13199 28474379

[70] Huang G Huang H. Hyaluronic acid-based biopharmaceutical delivery and tumor-targeted drug delivery system. J Control Release (2018) 278:122–6. doi: 10.1016/j.jconrel.2018.04.015 29649528

[71] Pali-Schöll I Szöllösi H Starkl P Scheicher B Stremnitzer C Hofmeister A. Protamine nanoparticles with CpG-oligodeoxynucleotide prevent an allergen-induced Th2-response in BALB/c mice. Eur J Pharm Biopharm (2013) 85:656–64. doi: 10.1016/j.ejpb.2013.03.003 23523543

[72] Nguyen QT Kwak C Lee WS Kim J Jeong J Sung MH. Poly-γ-glutamic acid complexed with alum induces cross-protective immunity of pandemic H1N1 vaccine. Front Immunol (2019) 10:1604. doi: 10.3389/fimmu.2019.01604 31354739 PMC6637289

[73] Calzada D Baos S Cremades L Cardaba B. New treatments for allergy: Advances in peptide immunotherapy. Curr Med Chem (2018) 25:2215–32. doi: 10.2174/0929867325666171201114353 29205109

[74] Komlósi ZI Kovács N Sokolowska M van de Veen W Akdis M Akdis CA. Highlights of novel vaccination strategies in allergen immunotherapy. Immunol Allergy Clin North Am (2020) 40:15–24. doi: 10.1016/j.iac.2019.09.010 31761116

[75] Jacquet A. Perspectives in allergen-specific Immunotherapy: Molecular evolution of peptide, and protein-based strategies. Curr Protein Pept Sci (2020) 21:203–23. doi: 10.2174/1389203720666190718152534 31416410

[76] Calzada D Cremades-Jimeno L López-Ramos M Cárdaba B. Peptide allergen immunotherapy: A new perspective in Olive-pollen allergy. Pharmaceutics (2021) 13:1007. doi: 10.3390/pharmaceutics13071007 34371699 PMC8309132

[77] Smith TRF Alexander C Kay AB Larche M Robinson DS. Cat allergen peptide immunotherapy reduces CD4+ T cell responses to cat allergen but does not alter suppression by CD4+ CD25+ T cells: a double-blind placebo-controlled study. Allergy (2004) 59:1097–101. doi: 10.1111/j.1398-9995.2004.00601.x 15355469

[78] Campbell JD Buckland KF McMillan SJ Kearley J Oldfield WLG Stern LJ. Peptide immunotherapy in allergic asthma generates IL-10–dependent immunological tolerance associated with linked epitope suppression. J Exp Med (2009) 206:1535–47. doi: 10.1084/jem.20082901 PMC2715096 19528258

[79] Alexander C Ying S B Kay A Larché M. Fel d 1-derived T cell peptide therapy induces recruitment of CD4+ CD25+; CD4+ interferon-gamma+ T helper type 1 cells to sites of allergen-induced late-phase skin reactions in cat-allergic subjects. Clin Exp Allergy (2005) 35:52–8. doi: 10.1111/j.1365-2222.2005.02143.x 15649266

[80] Larché M. Mechanisms of peptide immunotherapy in allergic airways disease. Ann Am Thorac Soc (2014) 11 Suppl 5:S292–6. doi: 10.1513/AnnalsATS.201402-090AW PMC5473681 25525735

[81] Akagi T Kaneko T Kida T Akashi M. Preparation and characterization of biodegradable nanoparticles based on poly(gamma-glutamic acid) with l-phenylalanine as a protein carrier. J Control Release (2005) 108:226–36. doi: 10.1016/j.jconrel.2005.08.003 16125267

[82] Broos S Lundberg K Akagi T Kadowaki K Akashi M Greiff L. Immunomodulatory nanoparticles as adjuvants and allergen-delivery system to human dendritic cells: Implications for specific immunotherapy. Vaccine (2010) 28:5075–85. doi: 10.1016/j.vaccine.2010.05.004 20478343

[83] Amna T Hassan MS El-Newehy MH Alghamdi T Abdulhameed MM Khil MS. Biocompati-bility computation of muscle cells on polyhedral oligomeric silsesquioxane-grafted polyurethane na-nomatrix. Nanomater (2021) 11(11):2966. doi: 10.3390/NANO11112966 PMC8620573 34835731

[84] Makadia HK Siegel SJ. Poly lactic-co-glycolic acid (PLGA) as biodegradable controlled drug delivery carrier. Polymers (Basel) (2011) 3:1377–97. doi: 10.3390/polym3031377 PMC3347861 22577513

[85] Jilek S Walter E Merkle HP Corthésy B. Modulation of allergic responses in mice by using biodegradable poly(lactide-co-glycolide) microspheres. J Allergy Clin Immunol (2004) 114:943–50. doi: 10.1016/j.jaci.2004.05.065 15480340

[86] Pereira MA Rebouças JdeS Ferraz-Carvalho RdeS de Redín IL Guerra PV Gamazo C. Poly(anhydride) nanoparticles containing cashew nut proteins can induce a strong Th1 and Treg immune response after oral administration. Eur J Pharm Biopharm (2018) 127:51–60. doi: 10.1016/j.ejpb.2018.02.011 29428795

[87] Garaczi E Szabó K Francziszti L Csiszovszki Z Lőrincz O Tőke ER. DermAll nanomedicine for allergen-specific immunotherapy. Nanomedicine (2013) 9:1245–54. doi: 10.1016/j.nano.2013.05.011 23747740

[88] Amna T Hassan MS Pandeya DR Khil M-S Hwang IH. Classy non-wovens based on animate L. gasseri-inanimate poly(vinyl alcohol): upstream application in food engineering. Appl Microbiol Biotechnol (2013) 97:4523–31. doi: 10.1007/s00253-012-4666-z 23306644

[89] Tasaniyananda N Chaisri U Tungtrongchitr A Chaicumpa W Sookrung N. Mouse model of cat allergic rhinitis and intranasal liposome-adjuvanted refined fel d 1 vaccine. PloS One (2016) 11:e0150463. doi: 10.1371/journal.pone.0150463 26954254 PMC4783078

[90] Aliu H Rask C Brimnes J Andresen T. Enhanced efficacy of sublingual immunotherapy by liposome-mediated delivery of allergen. Int J Nanomedicine (2017) 12:8377–88. doi: 10.2147/ijn.s137033 PMC5702530 29200850

[91] Rask C Brimnes J Andresen TL. Enhanced efficacy of sublingual immunotherapy by lipo-some-mediated delivery of allergen. Int J Nanomed (2017) 12:8377–88. doi: 10.2147/IJN.S137033 PMC5702530 29200850

[92] Kesharwani P Jain K Jain NK. Dendrimer as nanocarrier for drug delivery. Prog Polym Sci (2014) 39:268–307. doi: 10.1016/j.progpolymsci.2013.07.005

[93] Herzberger J Niederer K Pohlit H Seiwert J Worm M Wurm FR. Polymerization of ethylene oxide, propylene oxide, and other alkylene oxides: Synthesis, novel polymer architectures, and bioconjugation. Chem Rev (2016) 116:2170–243. doi: 10.1021/acs.chemrev.5b00441 26713458

[94] Pohlit H Bellinghausen I Schömer M Heydenreich B Saloga J Frey H. Biodegradable pH-sensitive poly(ethylene glycol) nanocarriers for allergen encapsulation and controlled release. Biomacromolecules (2015) 16:3103–11. doi: 10.1021/acs.biomac.5b00458 26324124

[95] Dumortier H. When carbon nanotubes encounter the immune system: desirable and undesirable effects. Adv Drug Deliv Rev (2013) 65:2120–6. doi: 10.1016/j.addr.2013.09.005 24056183

[96] Rodriguez MJ Ramos-Soriano J Perkins JR Mascaraque A Torres MJ Gomez F. Glycosylated nanostructures in sublingual immunotherapy induce long-lasting tolerance in LTP allergy mouse model. Sci Rep (2019) 9:4043. doi: 10.1038/s41598-019-40114-7 30858392 PMC6411722

[97] Ronzani C Casset A Pons F. Exposure to multi-walled carbon nanotubes results in aggravation of airway inflammation and remodeling and in increased production of epithelium-derived innate cytokines in a mouse model of asthma. Arch Toxicol (2014) 88:489–99. doi: 10.1007/s00204-013-1116-3 23948970

[98] Ryan JJ Bateman HR Stover A Gomez G Norton SK Zhao W. Fullerene nanomaterials inhibit the allergic response. J Immunol (2007) 179:665–72. doi: 10.4049/jimmunol.179.1.665 17579089

[99] Norton SK Wijesinghe DS Dellinger A Sturgill J Zhou Z Barbour S. Epoxyeicosatrienoic acids are involved in the C70 fullerene derivative–induced control of allergic asthma. J Allergy Clin Immunol (2012) 130:761–769.e2. doi: 10.1016/j.jaci.2012.04.023 22664166 PMC3955256

[100] Shershakova N Baraboshkina E Andreev S Purgina D Struchkova I Kamyshnikov O. Anti-inflammatory effect of fullerene C60 in a mice model of atopic dermatitis. J Nanobiotechnology (2016) 14. doi: 10.1186/s12951-016-0159-z PMC4727272 26810232

[101] Popescu R Grumezescu A. Metal based frameworks for drug delivery systems. Curr Top Med Chem (2015) 15:1532–42. doi: 10.2174/1568026615666150414145323 25877086

[102] Barreto E Serra MF Dos Santos RV Dos Santos CEA Hickmann J Cotias AC. Local administration of gold nanoparticles prevents pivotal pathological changes in Murine models of atopic asthma. J Biomed Nanotechnol (2015) 11:1038–50. doi: 10.1166/jbn.2015.2024 26353593

[103] De S Rebouças J Irache JM Camacho AI Gastaminza G Sanz ML Ferrer M. Immunogenicity of peanut proteins containing poly(anhydride) nanoparticles. Clin Vaccine Immunol (2014) 21:1106–12. doi: 10.1128/CVI.00359-14 PMC4135921 24899075

[104] Marengo M Bonomi F Iametti S Prinz E Hempelmann R Boye M. Recognition and uptake of free and nanoparticle-bound betalactoglobulin , a food allergen , by human monocytes. Mol Nutr Food Res (2011) 55:1708–16. doi: 10.1002/mnfr.201100386 21953745

[105] Srivastava KD Siefert A Fahmy TM Caplan MJ Li X-M Sampson HA. Investigation of peanut oral immunotherapy with CpG/peanut nanoparticles in a murine model of peanut allergy. J Allergy Clin Immunol (2016) 138:536–543.e4. doi: 10.1016/j.jaci.2016.01.047 27130858

[106] Roy K Mao HQ Huang SK Leong KW. Oral gene delivery with chitosan–DNA nanoparticles generates immunologic protection in a murine model of peanut allergy. Nat Med (1999) 5:387–91. doi: 10.1038/7385 10202926

[107] Liu Q Wang X Liao Y-P Chang CH Li J Xia T. Use of a liver-targeting nanoparticle platform to intervene in peanut-induced anaphylaxis through delivery of an Ara h2 T-cell epitope. Nano Today (2022) 42:101370. doi: 10.1016/j.nantod.2021.101370 36969911 PMC10038170

[108] Hu J Xu X Xu L Kuang H Xu C Guo L. Gold nanoparticle-based lateral flow immunoassay for the rapid and on-site detection of wheat allergen in milk. Food Bioscience (2023) 51:102353. doi: 10.1016/j.fbio.2023.102353

[109] Liu M Thijssen S Hennink WE Garssen J van Nostrum CF Willemsen LEM. Oral pretreatment with β-lactoglobulin derived peptide and CpG co-encapsulated in PLGA nanoparticles prior to sensitizations attenuates cow’s milk allergy development in mice. Front Immunol (2023) 13:1053107. doi: 10.3389/fimmu.2022.1053107 36703973 PMC9872660

