# Corrigendum: Modulation of immune response by nanoparticle-based immunotherapy against food allergens

**DOI:** 10.3389/fimmu.2023.1332906

**Published:** 2023-12-06

**Authors:** Sivadas Swathi Krishna, Syeda Ayesha Farhana, Ardra T.P., Shalam M. Hussain, Vidya Viswanad, Muhammed Hassan Nasr, Ram Kumar Sahu, Jiyauddin Khan

**Affiliations:** ^1^ Department of Pharmaceutics, Amrita School of Pharmacy, Amrita Institute of Medical Science (AIMS) Health Science Campus, Amrita Vishwa Vidyapeetham, Kochi, India; ^2^ Department of Pharmaceutics, Unaizah College of Pharmacy, Qassim University, Unaizah, Saudi Arabia; ^3^ Department of Clinical Pharmacy, College of Nursing and Health Sciences, Al-Rayyan Medical College, Madinah, Saudi Arabia; ^4^ Department of Clinical Pharmacy, Faculty of Health Sciences and Nursing, Al-Rayan Colleges, Al-Madinah Al-Munawarah, Saudi Arabia; ^5^ Department of Pharmaceutical Sciences, Hemvati Nandan Bahuguna Garhwal University (A Central University), Chauras, Tehri, Uttarakhand, India; ^6^ School of Pharmacy, Management and Science University, Shah Alam, Selangor, Malaysia

**Keywords:** allergen immunotherapy, food-induced anaphylaxis, oral immunotherapy, sublingual immunotherapy, nanoparticles

In the published article, there was an error in the affiliation of Syeda Ayesha Farhana. Instead of “Department of Pharmaceutics, Unaiza College of Pharmacy, Qassim University, Burayda, Al Qassim, Saudi Arabia”, it should be “Department of Pharmaceutics, Unaizah College of Pharmacy, Qassim University, Unaizah, Saudi Arabia”.

The authors apologize for this error and state that this does not change the scientific conclusions of the article in any way. The original article has been updated.

